# Unveiling the photophysical and morphological properties of an acidochromic thiophene flanked dipyrrolopyrazine-based chromophore for optoelectronic application†

**DOI:** 10.1039/c7ra12527e

**Published:** 2018-01-09

**Authors:** Puttavva Meti, Young-Dae Gong

**Affiliations:** Innovative Drug Library Research Center, Department of Chemistry, College of Science, Dongguk University 26, 3-ga, Pil-dong, Jung-gu Seoul 04620 Korea ydgong@dongguk.edu +82-2-2290-1349 +82-2-2260-3206

## Abstract

A series of dipyrrolopyrazine (DPP) based chromophores featuring thiophene and varied donor (*N*,*N*-dimethylamine, NH_2_, OMe) and acceptor (CF_3_, CN, NO_2_) appendages have been synthesized. The structures and properties of the chromophores were investigated by absorption spectroscopy, electrochemistry, differential scanning calorimetry, and thermogravimetric analysis. X-ray crystallographic analysis indicates a planar geometry for the molecule 7g. Surface morphological studies reveal the formation of microrods and nanorods. The acidochromic behavior of the chromophore which shows a prominent red-shift in the absorption spectra owing to the protonation of the pyrazine segment provides a valuable opportunity to assess the sensory response. Acid dependent spectral changes could be successfully applied to detect pH in biological fluids and acid impurities in solvents.

## Abstract 2

A series of dipyrrolopyrazine (DPP) based chromophores featuring thiophene and varied donor (*N*,*N*-dimethylamine, NH_2_, OMe) and acceptor (CF_3_, CN, NO_2_) appendages have been synthesized.

## Graphical abstract

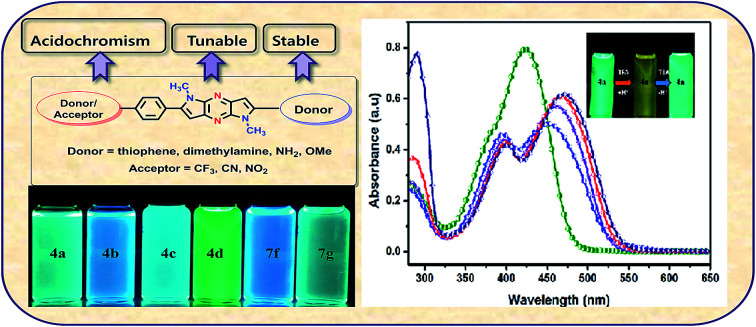

## 1. Introduction

In recent years, a large number of π-conjugated organic molecules, mainly push–pull chromophores, have received a lot of interest owing to their applications in a wide range of electronic and optoelectronic devices. One commonly used strategy to design π-electron chromophores is to end-terminate suitable conjugated bridges with strong electron donor and acceptor substituents.^1–7^ This D–π–A arrangement^8^ ensures efficient intramolecular charge transfer (ICT) between donor and acceptor and generates a dipolar push–pull system. The electronic and structural properties of chromophores with donor–acceptor (D–π–A) substituted organic compounds are of considerable interest because of their potential applications in nonlinear optical materials (NLO),^9–11^ light emitting diodes (OLED),^12,13^ and sensors^14^ as well as organic field effect transistors (OFET).^15–17^ Beside these widely used applications, push–pull chromophores are also used in organic photovoltaic cells (PVCs),^18^ dye sensitised solar cells (DSSC),^19–22^ bulk-heterojunction solar cells (BHJ),^23^ and biological imaging.^24,25^ Direct interaction in push–pull chromophores provides the π-conjugated molecule with additional properties such as dipolar character, intense colour, chemical and thermal robustness.^26^ Conversely, many organic materials like polyenes suffer from low thermal stability. Therefore extensive research efforts have been directed towards modification of donor, acceptor, and π-conjugated moieties.

Synthetic studies have shown that replacement of benzene ring with easily delocalizable five membered heteroaromatic ring (furan, pyrrole or thiophene)^27–32^ play vital role in determining the overall electron donating ability of the substituent. However, the pyrrole-based bridge, the analogue of thiophene moieties, has seldom been noticed. Pyrrole^33,34^ containing chromophores as the π-conjugated bridge were found to display good optical properties in comparison with their analogues with furan or thiophene. This was attributed to the higher electron density in the pyrrole moiety compared to thiophene and furan.

Thiophene have been used as efficient electron donor as it imparts an enhance ICT in the chromophore, thiophene is undoubtedly among most explored heterocyclic moiety and has already found many applications^35^ in organic electronic and photonics because of its high chemical and photophysical stability compared to the other heteroaromatics. Thiophene as a part of push–pull molecules has been investigated by Filip Bureš *et al.*^36^ and other groups. The pyrazine rings have also been extensively used as electron withdrawing part of push–pull systems.^37–42^ Linear chromophores incorporating pyrazine moiety exhibits interesting emission properties.^43^ Only a few examples of incorporating pyrazine rings as π-linker in push–pull system have been reported. However, to the best of our knowledge, thiophene flanked DPP chromophores have not been studied as a part of push–pull chromophores.

We have previously introduced π-conjugated DPP skeletal backbone^44–46^ and their photophysical properties. In this work, we decided to investigate the effect of presence and position of electron-rich and electron-deficient moieties on the photophysical properties. The most efficient charge-transfer between these two functionalities is achieved when they are attached to the C-2 and C-6 positions of the dipyrrolopyrazine. DPP was used as π-conjugated bridge functionalized with most powerful electron donor^47^ (*N*,*N*-dimethylamine, NH_2_, OMe) and acceptor^48,49^ (CF_3_, CN, NO_2_). CF_3_ and CN groups are introduced as they are expected to affect the molecular packing mode by hydrogen bonding interaction. Acid dependent fluorescent materials^50–52^ have been intensively studied in terms of practical applications in the field of sensors, memories, and display device. Thus we investigated the sensitivity of these chromophores toward acids to explore their feasibility towards sensor industry.

## 2. Results and discussion

### 2.1 Synthesis

**Scheme 1:**
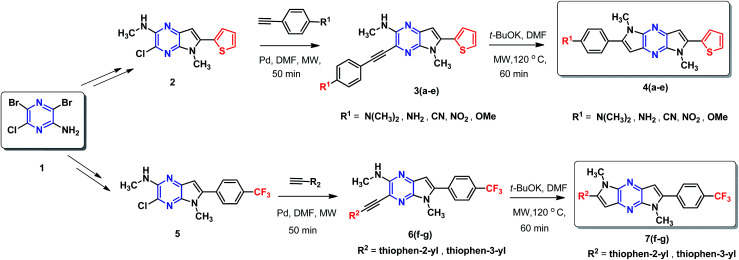
Synthetic scheme employed for the preparation of DPP-based chromophore.

A simple and efficient protocol was employed as outlined in Scheme 1. Extended 2-thiophene and trifluoromethane- substituted π-linkers 2 and 5, which are required for the synthesis of chromophores 4(a–e) and 7(f–g) were prepared in a modular manner.^45,46^ Synthesis of target chromophores was accomplished by two-step process. First C–C coupling *via* Sonogashira coupling reaction followed by intramolecular cyclization. PdCl_2_(PPh_3_)_2_ catalysed Sonogashira cross-coupling reaction between free amine (2,5) and acetylene containing electron withdrawing groups (CF_3_, CN, NO_2_) and electron donating groups (thiophen-2-yl, thiophen-3-yl, *N*,*N*-dimethylaniline, NH_2_, OMe) under microwave (MW) afforded C–C coupled product 3(a–e) and 6(f–g) in modest yield, along with small amount of cyclic product, separation of the mixture was tedious in some case so the mixture was directly used for next step. The reaction with 1-ethynyl-4-nitrobenzene and 4-ethynylbenzonitrile was sluggish and low-yielding, although the reaction conditions were optimised, increased temperature or reaction time led to decomposition.

Next, base induced intramolecular cyclization of 3(a–e) and 6(f–g) under MW condition furnished corresponding cyclic compounds 4(a–e) and 7(f–g) with moderate yield. Intramolecular cyclization of 3e afforded only traces of desired chromophores 4e. Purification of products was carried out by means of standard column chromatography. All the prepared chromophores are easily soluble in regular organic solvents such as chloroform, dichloromethane, methanol to give bluish-green solution and the compounds can be stored for long time without decomposition. The structures of prepared chromophores were unambiguously confirmed by their spectral and analytical data (Table 1).

**Table 1:** Structure of intermediate compound and cyclic chromophores

No	product	Yield (%)	No	product	Yield (%)
3a	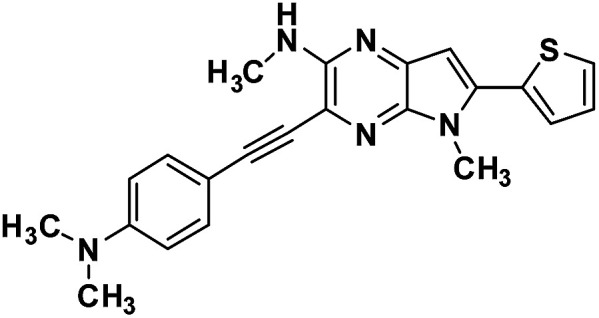	42^a^	4a	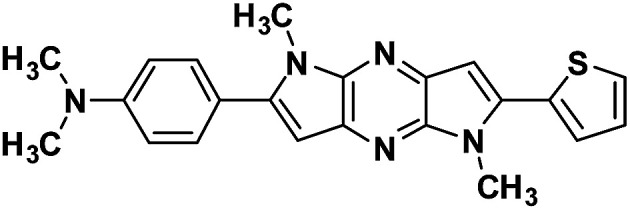	75
3b	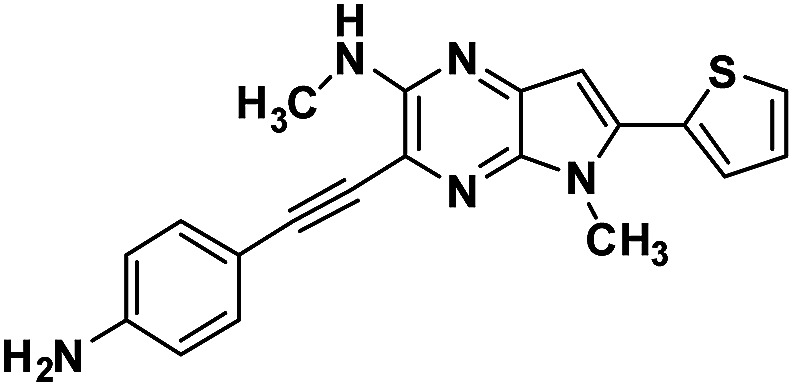	67^a^	4b	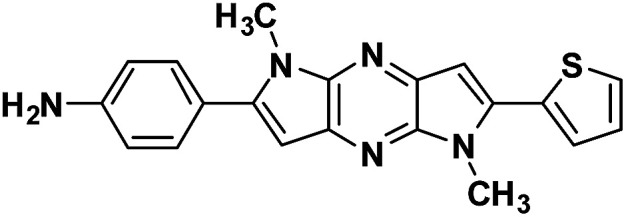	53
3c	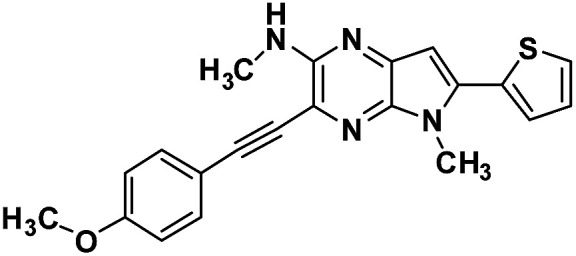	64^a^	4c	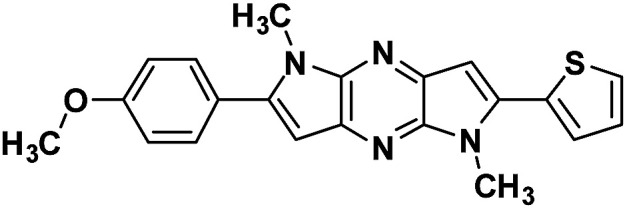	81
3d	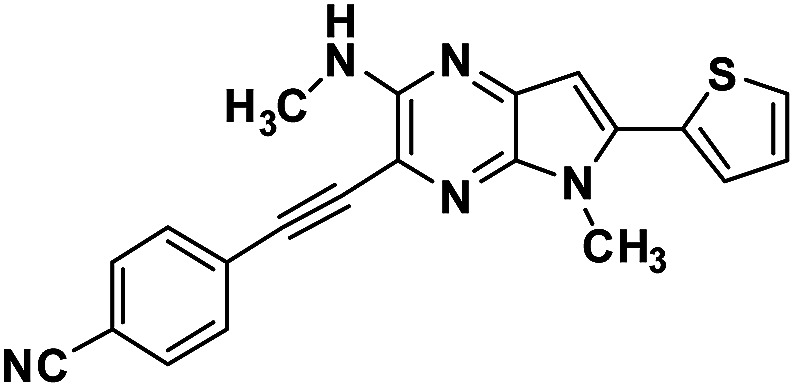	48	4d	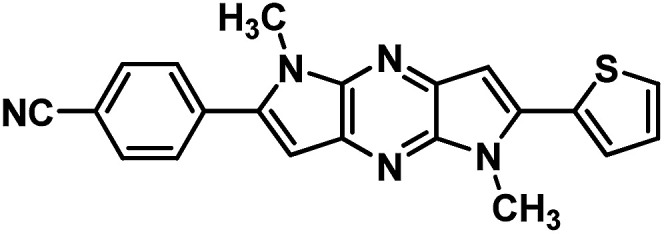	62
6f	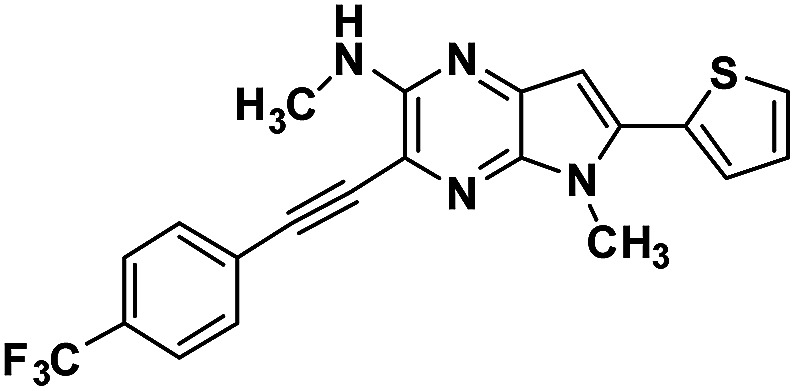	58^a^	7f	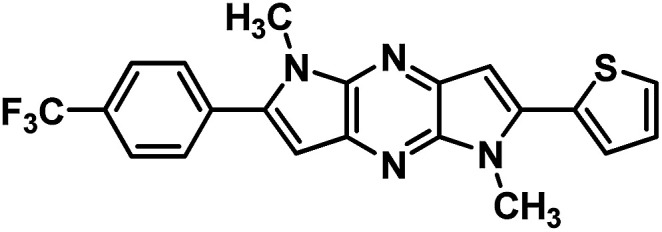	82
6g	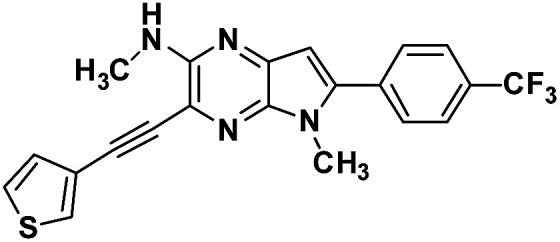	63	7g	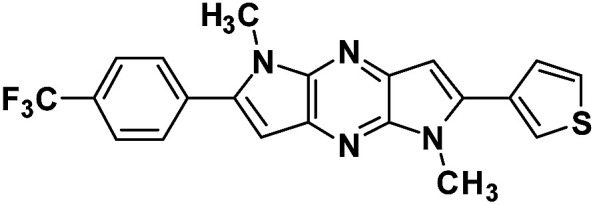	71

a Trace amount of cyclization product was also observed.

### 2.2 Crystallographic analysis

**Fig. 1:**
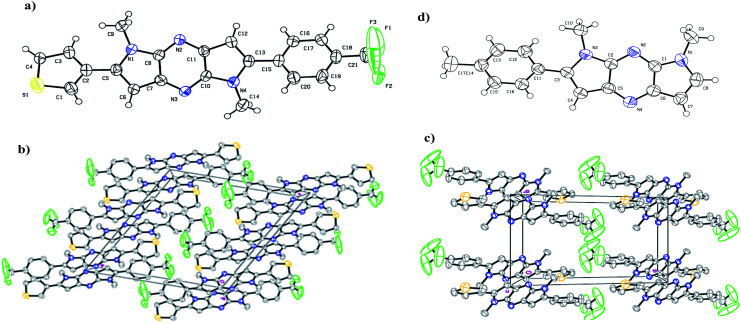
(a) ORTEP representation and spatial arrangement of compound 7g. (b) Molecular packing diagram viewed along a axis (c) packing along *c* axis. (d) ORTEP representation of 1,7-dimethyl-2-*p*-tolyl-1,7-dihydrodipyrrolo[2,3-*b*:3′,2′-*e*]pyrazine showing slight twist of the phenyl ring.

The X-ray crystallography was performed to investigate solid-state packing and the interactions in the chromophores. In our previous work,^44^ we have shown the non planar arrangement in 1,7-dimethyl-2-*p*-tolyl-1,7-dihydrodipyrrolo[2,3-*b*:3′,2′-*e*]pyrazine which is barrier for effective D–A conjugation. Thus we prepared chromophores possessing D and A connected to π-linker (DPP) through C-2 and C-6 position. This arrangement assured planarization of the entire π-conjugated system. Single crystal suitable for structural analysis was obtained by recrystallization from DCM. The illustration of crystal packing for 7g is shown in Fig. 1. X-ray crystallographic analysis indicates planar geometry of the molecules with π-stacking, which facilitates ICT process through the molecule which in turn finely tunes the absorption. Compound 7g crystallised in the monoclinic system with space group *P*2(1)/*c*, and the unit cell dimensions of (a) 15.07 (2) Å, (b) 8.80 (10) Å, (c) 15.41 (2) Å, *α* = 90°, *β* = 115.2(10)°, and *γ* = 90°.

## 3. Photophysical properties

### 3.1 Optical properties

**Fig. 2:**
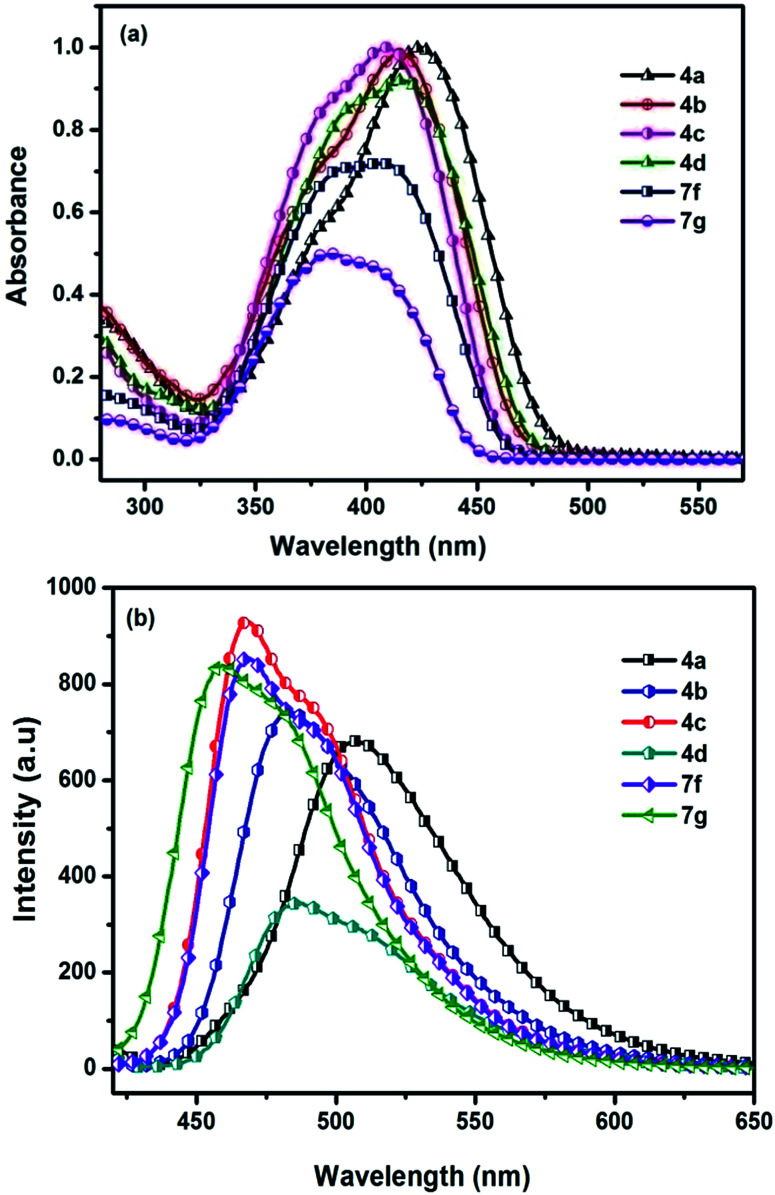
(a) Absorption spectra (b) emission spectra of chromophores 4(a–d) and 7(f–g) recorded in DCM.

**Fig. 3:**
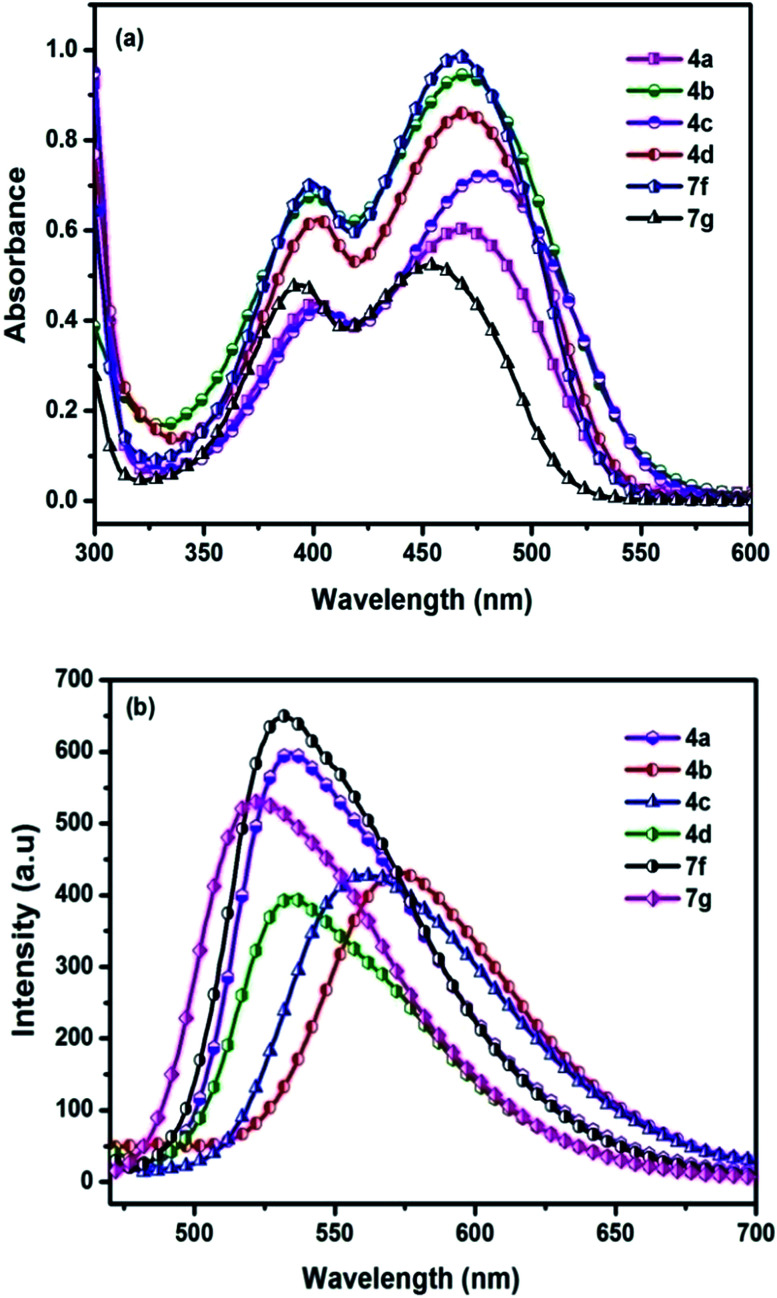
(a) Absorption spectra (b) emission spectra of chromophores recorded in DCM after addition of TFA.

Absorption spectra of the chromophores were recorded in DCM solution. All of the chromophores exhibited a broad intense absorption band (Fig. 2(a)). The position of the absorption bands is influenced by the functional groups of the compound and assumes a trend in the order (7g < 7f < 4c < 4d < 4b < 4a) reflecting the impact of donor moiety. Chromophores with electron donating group exhibit bathochromic shift due to involvement of conjugative delocalization. Extended conjugation present in these derivatives lead to red shift profile. On the contrary, blue shift profile observed in chromophores with electron withdrawing groups. So compound 4a possesses largest wavelength and 7g possesses lowest. The absorption and emission of 7g are small which may be attributed to lower intramolecular charge transfer. The optical band gap values were approximated from the onset of the absorption spectra and the relevant parameters are compiled in Table 2. Solvatochromism for the D–A molecules originate due to the difference in the dipole moment. Chromophore 4a studied in this work exhibited slight red shift when recorded in polar solvents indicating negligible intramolecular interactions in the ground state. A representative variation of absorption spectra with different solvents is illustrated in Fig. 3(a).

**Table 2:** Optical and electronic properties of chromophores

Chromophore	*λ*_abs_ a [nm]		*λ*_em_ b [nm]		Cyclic voltammetry			
	DCM	DCM + TFA	DCM	DCM + TFA	*E* ^opt^_g_ c [eV]	*E*_ox_ d [eV]	HOMO^e^ [eV]	LUMO^f^ [eV]
4a	423	401, 471	507	534	2.55	0.56	−5.36	−2.81
4b	416	401, 470	483	575	2.60	0.65	−5.45	−2.85
4c	408	401, 479	468	560	2.64	0.71	−5.51	−2.87
4d	414	402, 471	488	535	2.58	0.90	−5.70	−3.12
7f	406	398, 466	470	531	2.66	0.91	−5.71	−3.05
7g	390	394, 454	461	520	2.77	0.95	−5.75	−2.98

a Absorption spectra.

b Emission spectra, both recorded in DCM before and after addition of TFA.

c Optical band gap was calculated from the UV-vis absorption onset in solution.

d Onset voltage of the first oxidation potential.

e HOMO levels of the compounds were determined from onset voltage of the first oxidation potential with reference to ferrocene using HOMO = −(*E*_ox_ + 4.8) eV.

f LUMO levels were estimated from the optical band gaps and the HOMO energy levels.

Fluorescence spectra of the compounds displayed moderately intense emission spectra in DCM solution when excited at their absorption maxima. The most red-shifted emission profile was observed for amine derivative (4a) while the thiophen-3-yl (7g) displayed the shorter wavelength emission.

Representative illustrations showing the emission profile are displayed in Fig. 2(b). Emission spectra of the chromophores were also examined in a series of solvents with varying polarity index to identify the impact of the polarity of the solvent on the excited state of the chromophores. For the chromophore 4a representative illustrations showing the influence of the solvent polarity on the emission profile are displayed in Fig. 1 (ESI data†). The emission profile of the chromophores exhibited a positive solvatochromism with the bathochromically shifted emission maxima in the polar solvents such as DMF, DMSO, and MeOH. Chromophores showed different types of interactions with the nonpolar and polar solvents. This suggests that less polar solvents, solvation effect is present while for the polar solvents additional specific interactions such as dipole–dipole relaxation plays a major role.

### 3.2 Acidochromism

**Fig. 4:**
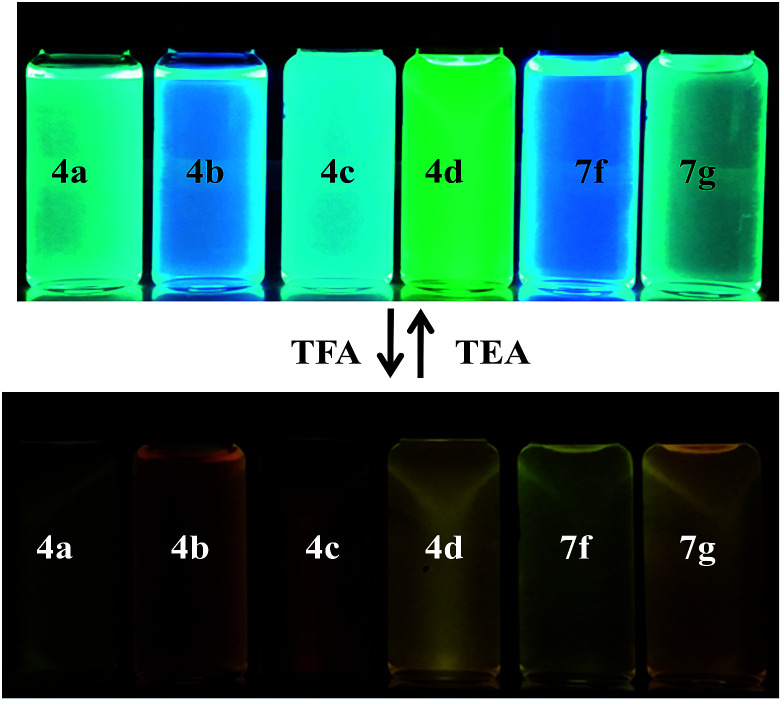
Photographic images of chromophores showing color change before and after addition of TFA under UV lamp (*λ*_em_ = 365 nm).

Another interesting phenomenon of the present chromophores was its fluorescent behavior stimulated by acid (acidochromism). Addition of CF_3_COOH (TFA) to the chromophores elicits a red-shift in both absorption and emission and the corresponding spectra are shown in Fig. 4. Similarly a sharp decrease in the emission intensity was noticed on the addition of TFA to DCM solutions with bathochromic shift, due to dipolar relaxation of D–A interactions from the excited state.^53^

**Scheme 2:**
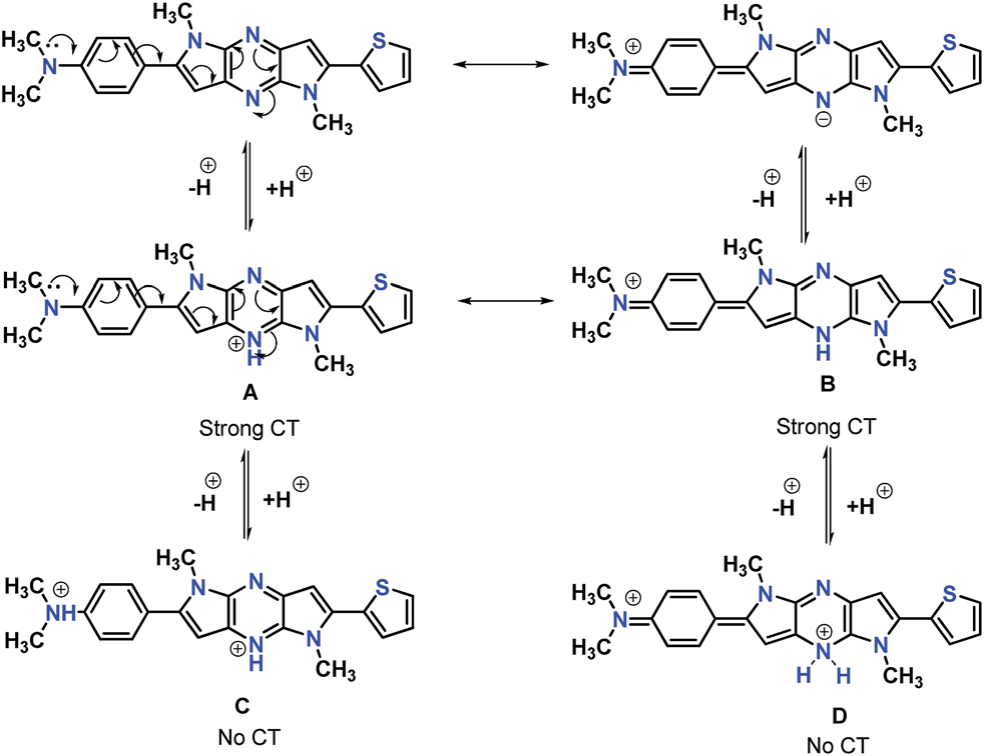
Protonation–deprotonation equilibrium of the chromophores in presence of TFA.

**Fig. 5:**
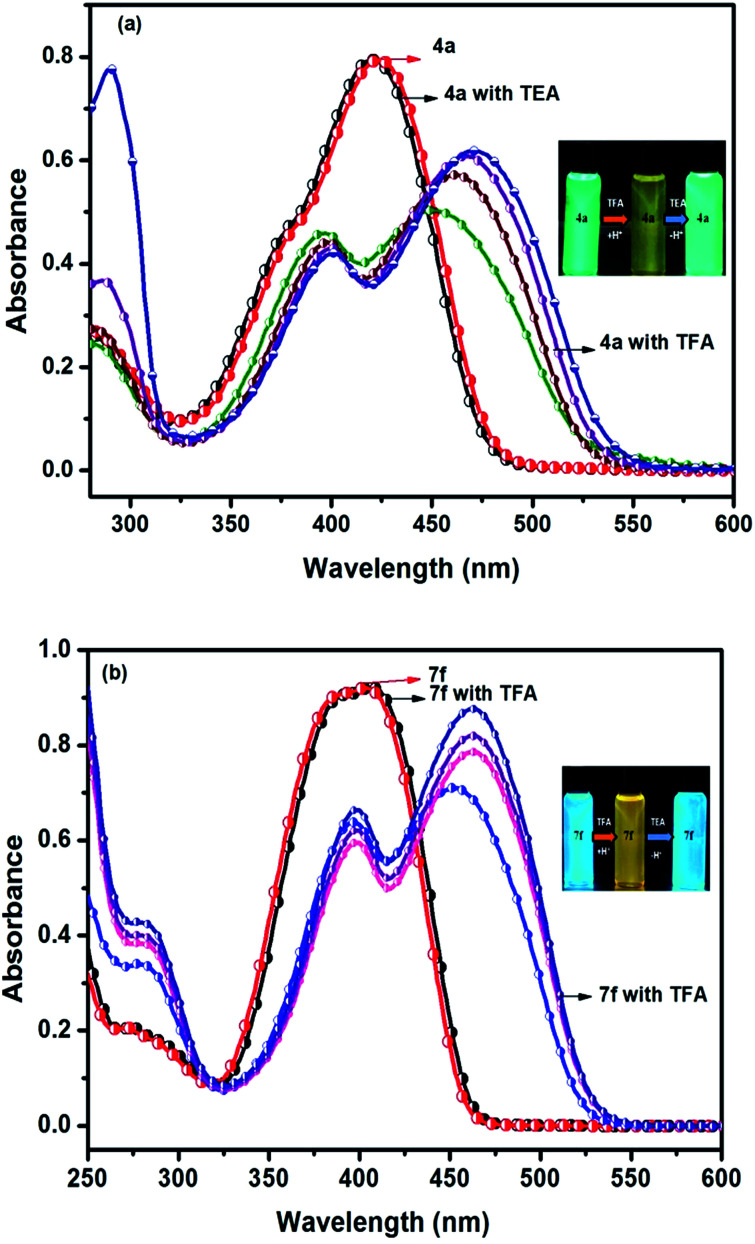
Reversible spectral changes in chromophores 4a and 7f recorded in DCM with incremental addition of TFA (10–40 equiv.).

In the absorption spectra of selected chromophores 4a (D–π–linker–D) and 7f (A–π–linker–D) the higher wavelength band was progressively red-shifted giving two main fluorescent peaks, with a color change from green (blue) to yellow. Similar effects were observed in the emission spectra. These changes could be ascribed to the protonation effect of TFA. The proton induced shifts in all chromophores are reminiscent.^54^ The pyrazine segment is protonated to generate pyrazinium ion, which trigger ICT between D–A (Scheme 2). Consequently, chromophores 4a and 7f are consanguine, pyrazine and thiophene core together dictates their absorption behavior. A representative illustration of changes for 4a and 7f in the absorption profile on incremental addition of TFA is shown in Fig. 5. In DCM solution, color of chromophores changes from green to yellowish as shown in Fig. 4. Photographic image of chromophores under normal light are shown in ESI data† (Fig. 2). Interestingly, adding triethylamine (TEA) to this system could restore its initial green (blue) state which neutralizes the effect arising due to the addition of TFA. The observation of isobestic points suggests the presence of neutral and protonated forms in equilibrium. These chromophores exhibit acid–base equilibrium as illustrated in Scheme 2.

### 3.3 Electrochemical properties

**Fig. 6:**
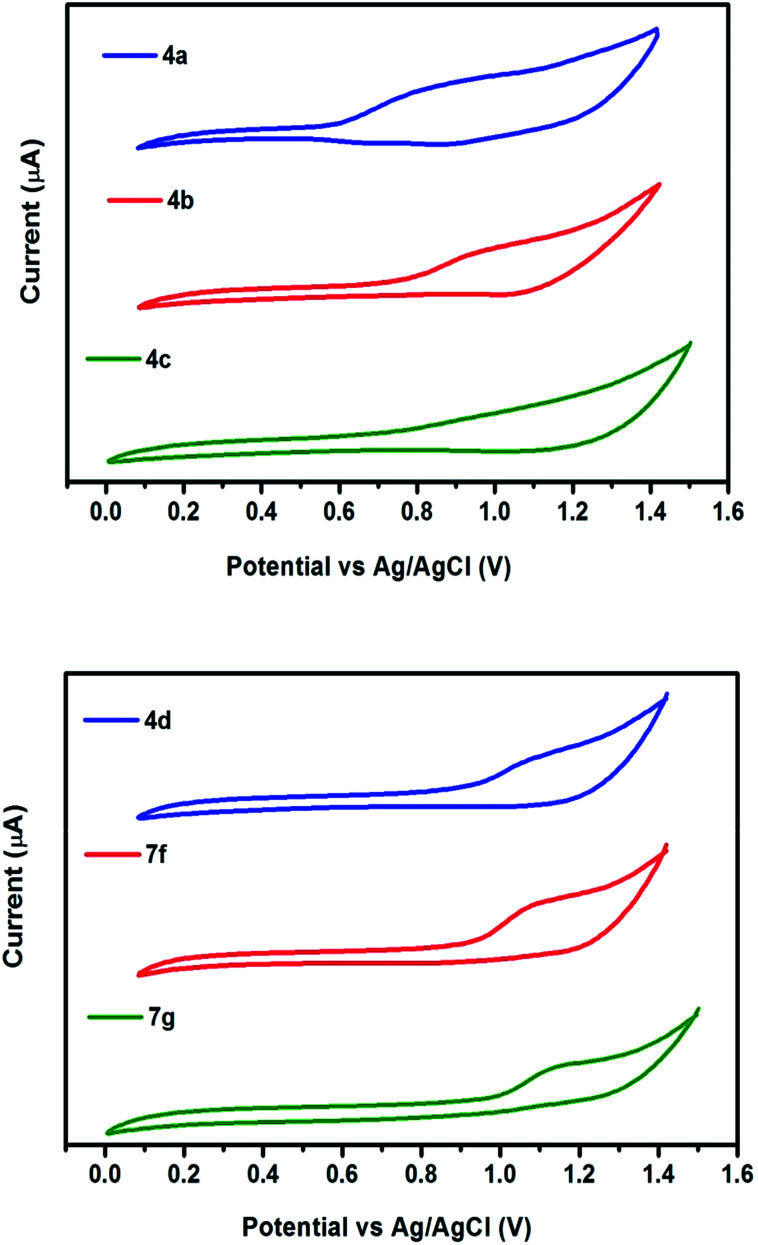
Cyclic voltammograms of chromophores recorded in DCM at a scan rate of 50 mV s^−1^.

The redox behavior of the chromophores 4(a–d) and 7(f–g) were scrutinized by cyclic voltammetry and their redox potentials and energy levels are shown in Table 2. The highest occupied molecular orbital (HOMO) energy levels, estimated from the oxidation onset. The LUMO levels are estimated from the HOMO value and the optical bandgap. The electronic interaction of donor and acceptor moieties affects the oxidation potentials. The donors interact effectively with acceptor moiety and lead to a reduction in the band gap. Thus lower oxidation potentials are accepted for the strong donor containing chromophores 4a and 4b. While larger oxidation potentials observed in 7g (Fig. 6). In agreement with these generalizations, the oxidation potentials of the chromophores assumed the order: 4a < 4b < 4c < 4d < 7f < 7g.

### 3.4 Thermal properties

**Fig. 7:**
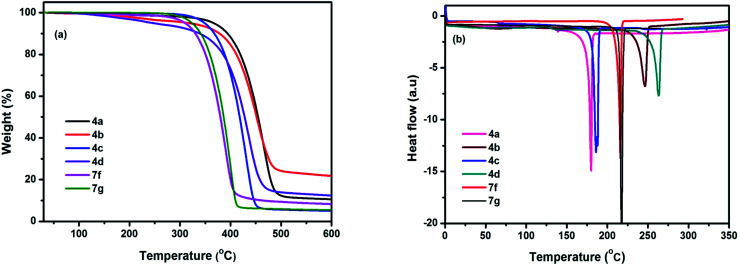
(a) TGA and (b) DSC plots of chromophores measured at a heating rate of 10 °C min^−1^ under nitrogen atmosphere.

Thermal behaviour of chromophores 4(a–d) and 7(f–g) were studied by thermogravimetric analysis (TGA) and differential scanning calorimetry (DSC). All the chromophores exhibited good thermal stabilities with the decomposition temperature (*T*_d_) higher than 300 °C under nitrogen atmosphere. The highest thermal stability observed in 4a, while 4d displayed the lowest in the series. This may be due to the different properties of modified groups (NMe_2_, NH_2_, OMe, CF_3_). The data indicate that the modified groups can influence the thermal stabilities of chromophores (Table 3). The phase transitions have been studied by DSC under nitrogen with a heating rate of 10 °C min^−1^ in the temperature range of 30–700 °C, neither phase transition nor thermal decomposition was observed upto 180 °C (Fig. 7). Sharp endothermic melting peak observed in the range 180–264 °C, indicates highly crystalline nature of the compounds. As determined by DSC and TGA experiments, compounds 4(a–d) and 7(f–g) has remarkable thermal stability for an organic material.

**Table 3:** Thermal properties of Chromophores

Chromophores	*T*_d_ a [°C]	*T*_m_ b [°C]
4a	340	180
4b	338	248
4c	364	187
4d	305	264
7f	310	216
7g	330	218

a Degradation temperature (*T*_d_) observed from TGA corresponding to 5% weight loss at 10 °C min^−1^ under nitrogen.

b Melting temperature (*T*_m_) from DSC at 10 °C min^−1^ under nitrogen.

### 3.5 X-ray diffraction

**Fig. 8:**
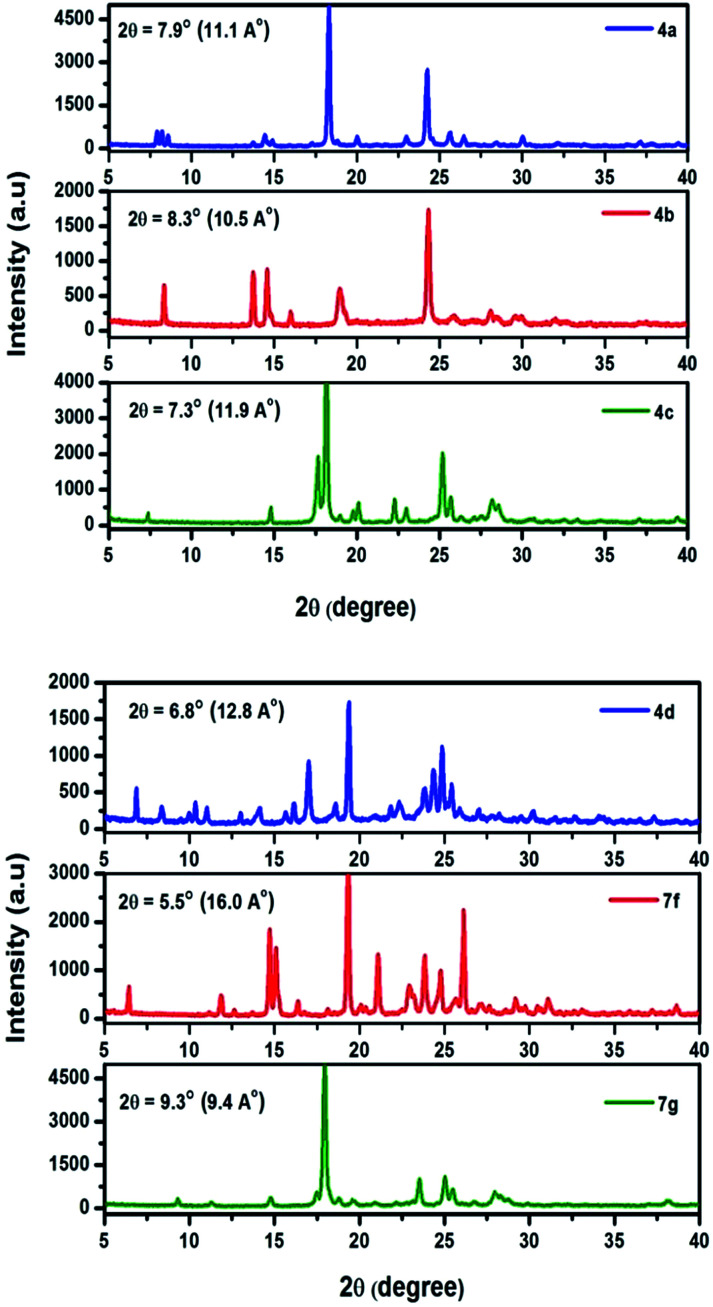
Powder X-ray diffraction pattern of 4(a–d) and 7(f–g).

X-ray diffraction pattern of prepared chromophores are displayed in Fig. 8. All of the chromophores exhibit strong diffraction intensities attributed to π–π interactions. Compound 4a exhibits a primary diffraction peak at 2*θ* = 7.9° corresponding to a *d*-spacing of 11.1 Å. Chromophore 4b, 4c, and 4d exhibit primary diffraction peaks at 2*θ* = 8.3° (*d*-spacing 10.5 Å), 7.3° (*d*-spacing 11.9 Å), and 6.8° (*d*-spacing 12.8 Å). Similarly the X-ray diffraction pattern of chromophores 7f and 7g exhibits a primary diffraction peak at 2*θ* = 5.5° (*d*-spacing 16.0 Å) and 9.3° (*d*-spacing 9.4 Å). This indicates that chromophores easily form well resolved molecular structure and well defined diffraction peaks indicating a high degree of crystallinity.

### 3.6 Surface morphology

**Fig. 9:**
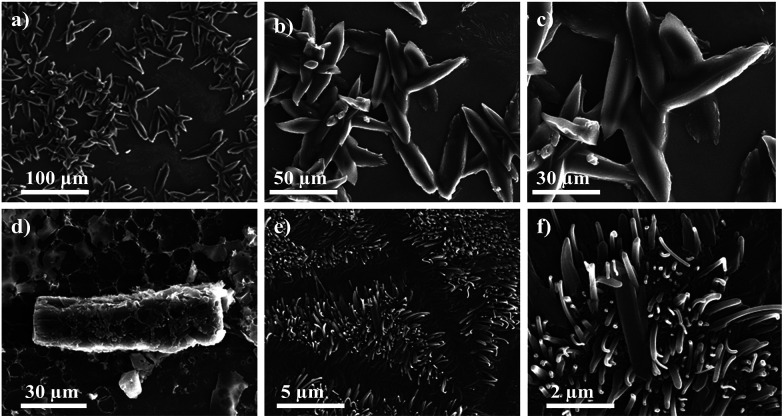
SEM images of self-assembled clusters of (a–c) 4a, (d) 4b, and (e and f) 4c, precipitated from its hot solution of DCM and THF, at different magnifications.

The morphology of thin film was also studied by scanning electron microscopy (SEM) in order to obtain more information about the thin-film forming properties of these chromophores. Thin films were prepared from drop casting THF and DCM solution of 4(a–d) and 7(f–g) on bare silica substrate followed by drying in air. SEM analysis reveals that compound 4(a–b) forms microrods and nanorods with an average length of 76 μm with thickness of 3 μm and 72 × 18 μm, respectively. Chromophores 4(c–d) also form rod like microstructures as observed in Fig. 9.

**Fig. 10:**
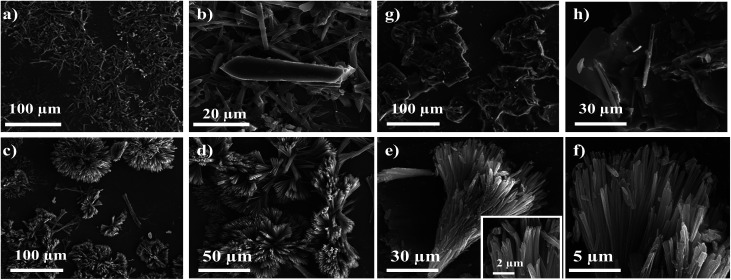
Typical morphologies of chromophores at different magnifications. SEM images of self-assembled microrods of (a and b) 4d, (c–f) 7g, and (g and h) 7f, precipitated from its hot solution of DCM.

SEM image of 7f reveals “chrysanthemum” like morphology, these flower-like supramolecular architectures is 60–70 μm in diameter, constructed from nanobelts with a thickness of 300–500 nm and width of 3–5 μm. Compound 7g form square facet with typical length-to-width ratio of the facts was about 10 μm (Fig. 10). The major driving force for formation of their supramolecular assemblies is the dipole–dipole interaction. The morphologies of the microstructures can be tuned by changing solvents. SEM image reveals well-ordered particles with 1-D and 3-D microstructure indicating highly crystalline nature with diverse particle size.

## 4. Experimental section

### 4.1 General methods

Solvents and reagents were purchased of reagent grade and used without further purification. ^1^H NMR and ^13^C NMR spectra were recorded on a Bruker 500 MHz NMR instrument. The chemical shifts were reported as *δ* (ppm) relative to a deuterated solvent as an internal reference and coupling constants (*J*) are reported in hertz (Hz). High-resolution mass spectrometry was performed using a 6550 iFunnel Q-TOF LC/MS system. Melting points were obtained from DSC thermograms. UV-vis and fluorescence spectra were recorded using standard 1 cm quartz cells on Varian cary-50 spectrophotometer and Cary Eclipse Fluorescence Spectrophotometer (excitation slit 5 nm). The spectra were recorded by using freshly prepared dilute solution. Compounds were excited at their absorption maxima. TGA and DSC were performed with TGA 3 plus (mettlertoledo) and DSC 2 STAR system (mettlertoledo) respectively, under nitrogen with heating rate of 10 °C min^−1^.

Cyclic voltammetry experiments were performed with ZIVE-SP2 LAB. All measurements were carried out at room temperature with a conventional three-electrode configuration consisting of a platinum working electrode, a platinum wire auxiliary, and a Ag/AgCl reference electrode. Ferrocene was used as an internal standard at a scan rate of 50 mV s^−1^. The solvent in all experiments was DCM, and the supporting electrolyte was 0.1 M tetrabutylammonium hexafluorophosphate. Single-crystal X-ray diffraction data were collected on Bruker SMART APEX II X-ray crystallography with CCD area detector SHELXL structural analysis program. Structure solution was done by direct method and refined by a full-matrix least-square method on *F*^2^. Powder XRD were analysed with Smartlab using Cu Kα radiation (*λ* = 1.5406 Å). SEM images were taken from FEI Quanta 3D FEG scanning electron microscope.

### 4.2 General method: sonogashira cross-coupling reaction

#### *N*,5-dimethyl-3-(phenylethynyl)-6-(thiophen-2-yl)-5*H*-pyrrolo[2,3-*b*]pyrazin-2-amine derivatives

Ethynyl substituted pyrrolopyrazine (2) (1.0 mmol) was dissolved in DMF (3 mL). TEA (3.0 mmol), CuI (0.1 mmol), Pd(PPh_3_)_2_Cl_2_ (0.1 mmol) and corresponding acetylene derivatives (1.0 mmol) were added subsequently. The mixture is subjected to MW condition for an hour at 90 °C. After cooling the reaction mixture was diluted with water and extracted with EA (3 × 20 mL). The combined organic layers was concentrated in vacuum and dried over MgSO_4_, and solvent was evaporated. The resulting residue was purified by column chromatography (25% EA: hexane).

#### 3-((4-(dimethylamino)phenyl)ethynyl)-*N*,5-dimethyl-6-(thiophen-2-yl)-5*H*-pyrrolo[2,3-*b*]pyrazin-2-amine (3a)

Brown solid. HRMS (ESI) *m*/*z*: [M + H]^+^ calculated for C_22_H_22_N_5_S, 388.1590; found 388.1599.

#### 3-((4-aminophenyl)ethynyl)-*N*,5-dimethyl-6-(thiophen-2-yl)-5*H*-pyrrolo[2,3-*b*]pyrazin-2-amine (3b)

Dark brown solid. HRMS (ESI) *m*/*z*: [M + H]^+^ calculated for C_22_H_18_N_5_S, 360.1277; found 360.1283.

#### 3-((4-methoxyphenyl)ethynyl)-*N*,5-dimethyl-6-(thiophen-2-yl)-5*H*-pyrrolo[2,3-*b*]pyrazin-2-amine (3c)

Brown solid. HRMS (ESI) *m*/*z*: [M + H]^+^ calculated for C_21_H_19_N_4_OS, 375.1274; found 375.1280.

#### [2,3-*b*]pyrazin-3-yl)ethynyl)benzonitrile (3d)

Orange crystals. ^1^H NMR (500 MHz, CDCl_3_) *δ* 7.68 (d, *J* = 8.3 Hz, 2H), 7.65 (d, *J* = 8.3 Hz, 2H), 7.46 (d, *J* = 5.1 Hz, 1H), 7.36 (d, *J* = 3.5 Hz, 1H), 7.18–7.15 (m, 1H), 6.63 (s, 1H), 5.20 (d, *J* = 4.8 Hz, 1H), 3.94 (s, 3H), 3.13 (d, *J* = 4.9 Hz, 3H). ^13^C NMR (126 MHz, CDCl_3_) *δ* 153.4, 139.4, 137.2, 137.2, 133.2, 132.1, 132.0, 128.0, 127.4, 127.2, 127.2, 118.3, 116.5, 111.9, 99.4, 93.9, 90.4, 29.9, 28.7. HRMS (ESI) *m*/*z*: [M + H]^+^ calculated for C_21_H_16_N_5_S, 370.1121; found 370.1134.

#### *N*,5-dimethyl-3-((4-nitrophenyl)ethynyl)-6-(thiophen-2-yl)-5*H*-pyrrolo[2,3-*b*]pyrazin-2-amine (3e)

Back solid. ^1^H NMR (500 MHz, CDCl_3_) *δ* 8.25 (d, *J* = 8.7 Hz, 2H), 7.75 (d, *J* = 8.7 Hz, 2H), 7.48 (d, *J* = 5.1 Hz, 1H), 7.38 (d, *J* = 3.3 Hz, 1H), 7.20–7.17 (m, 1H), 6.64 (s, 1H), 5.19 (d, *J* = 4.7 Hz, 1H), 3.95 (s, 3H), 3.15 (d, *J* = 4.9 Hz, 3H). ^13^C NMR (126 MHz, CDCl_3_) *δ* 153.5, 147.2, 139.7, 137.4, 137.3, 133.2, 132.3, 129.2, 128.0, 127.5, 127.3, 123.7, 116.3, 99.5, 93.8, 91.4, 29.9, 28.7. HRMS (ESI) *m*/*z*: [M + H]^+^ calculated for C_20_H_16_N_5_O_2_S, 390.1019; found 390.1027.

#### *N*,5-dimethyl-3-(thiophen-2-ylethynyl)-6-(4-trifluoromethyl) phenyl-5*H*-pyrrolo[2,3-*b*]pyrazin-2-amine (6f)

The title compound was synthesised from 5*via* Sonogashira reaction. Yellow crystals. ^1^H NMR (500 MHz, CDCl_3_) *δ* 7.76 (d, *J* = 8.1 Hz, 2H), 7.68 (d, *J* = 8.1 Hz, 2H), 7.41 (d, *J* = 3.4 Hz, 1H), 7.38 (d, *J* = 5.1 Hz, 1H), 7.07–7.03 (m, 1H), 6.58 (s, 1H), 5.25 (d, *J* = 4.7 Hz, 1H), 3.83 (s, 3H), 3.14 (d, *J* = 4.9 Hz, 3H). ^13^C NMR (126 MHz, CDCl_3_) *δ* 153.34, 143.75, 137.1, 136.5, 135.3, 133.1, 129.3, 128.5, 127.3, 125.7 (q, *J* = 3.7 Hz), 125.1, 122.9, 122.0, 118.3, 100.5, 89.0, 30.0, 28.7. HRMS (ESI) *m*/*z*: [M + H]^+^ calculated for C_21_H_16_F_3_N_4_S, 413.1042; found 413.1053.

#### *N*,5-dimethyl-3-(thiophen-3-ylethynyl)-6-(4-trifluoromethyl) phenyl-5*H*-pyrrolo[2,3-*b*]pyrazin-2-amine (6g)

The title compound was synthesised from 5*via* Sonogashira cross-coupling reaction. Pale yellow powder. ^1^H NMR (500 MHz, CDCl_3_) *δ* 7.75 (d, *J* = 7.8 Hz, 2H), 7.68 (d, *J* = 7.5 Hz, 2H), 7.65 (s, 1H), 7.34 (d, *J* = 1.8 Hz, 1H), 7.29 (d, *J* = 4.5 Hz, 1H), 5.27 (s, 1H), 3.83 (s, 3H), 3.14 (d, *J* = 4.4 Hz, 3H). ^13^C NMR (126 MHz, CDCl_3_) *δ* 153.2, 143.4, 137.2, 136.33, 135.4, 130.5, 130.3, 129.9 (d, *J* = 15.5 Hz), 129.2 (d, *J* = 15.5 Hz), 125.7 (q, *J* = 3.7 Hz), 125.0, 122.9, 121.2, 118.8, 100.3, 91.2, 85.3, 30.0, 28.7. HRMS (ESI) *m*/*z*: [M + H]^+^ calculated for C_21_H_16_F_3_N_4_S, 413.1042; found 413.1048.

### 4.3 General procedure for intramolecular cyclization

#### 1,5-Dimethyl-2-phenyl-6-(thiophen-2-yl)-1,5-dihyrodipyrrolo[3,2-*b*:3′,2′-*e*]pyrazine derivative (4a–e) and (7f–g)

To a stirred solution of acyclic compound (1.3 mmol) in DMF (4 mL) *t*-BuOK (2.7 mmol) was added. The resultant solution was refluxed at 120 °C for 60 min under MW. Reaction mixture was cooled to ambient temperature then diluted with DCM and water, organic layer washed with water and dried over anhydrous MgSO_4_, filtered and concentrated to furnish the crude compound which is purified by column chromatography (30% EA: Hexane).

#### 4-(1,5-Dimethyl-6-(thiophen-2-yl)-1,5-dihyrodipyrrolo[3,2-*b*:3′,2′-*e*]pyrazin-2-yl)-*N*,*N*-dimethyl aniline (4a)

Orange powder. ^1^H NMR (500 MHz, CDCl_3_) *δ* 7.50 (d, *J* = 8.5 Hz, 2H), 7.43 (d, *J* = 4.9 Hz, 1H), 7.36 (d, *J* = 3.1 Hz, 1H), 7.20–7.14 (m, 1H), 6.86 (s, 1H), 6.82 (d, *J* = 8.5 Hz, 2H), 6.65 (s, 1H), 4.06 (s, 3H), 3.94 (s, 3H), 3.03 (s, 6H). ^13^C NMR (126 MHz, CDCl_3_) *δ* 150.4, 146.1, 142.1, 141.5, 136.2, 135.8, 134.1, 133.6, 129.9, 127.8, 126.6, 126.6, 119.6, 112.0, 99.6, 97.6, 40.3, 30.1, 29.9. HRMS (ESI) *m*/*z*: [M + H]^+^ calculated for C_22_H_22_N_5_S, 388.1590; found 388.1579.

#### 4-(1,5-Dimethyl-6-(thiophen-2-yl)-1,5-dihyrodipyrrolo[3,2-*b*:3′,2′-*e*]pyrazin-2-yl)aniline (4b)

Brown solid. ^1^H NMR (500 MHz, CDCl_3_) *δ* 7.44 (dd, *J* = 5.1, 0.9 Hz, 1H), 7.43–7.40 (m, 2H), 7.37 (dd, *J* = 3.6, 0.9 Hz, 1H), 7.18 (dd, *J* = 5.1, 3.7 Hz, 1H), 6.86 (s, 1H), 6.80 (d, *J* = 8.5 Hz, 2H), 6.65 (s, 1H), 4.07 (s, 3H), 3.92 (s, 3H). ^13^C NMR (126 MHz, CDCl_3_) *δ* 146.9, 145.8, 142.0, 141.6, 136.4, 135.6, 134.0, 133.9, 130.3, 127.9, 126.7, 122.1, 114.9, 114.6, 99.6, 97.9, 30.0, 29.9. HRMS (ESI) *m*/*z*: [M + H]^+^ calculated for C_20_H_18_N_5_S, 360.1277; found 360.1270.

#### 2-(4-methoxyphenyl)-1,5-dimethyl-6-(thiophen-2-yl)-1,5-dihyro dipyrrolo[3,2-*b*:3′,2′-*e*]pyrazine (4c)

Light brown crystals. ^1^H NMR (500 MHz, CDCl_3_) *δ* 7.56 (d, *J* = 8.6 Hz, 2H), 7.45 (dd, *J* = 5.1, 0.9 Hz, 1H), 7.38 (dd, *J* = 3.6, 0.9 Hz, 1H), 7.21–7.17 (m, 1H), 7.05 (d, *J* = 8.7 Hz, 2H), 6.87 (s, 1H), 6.68 (s, 1H), 4.08 (s, 3H), 3.93 (s, 3H), 3.89 (s, 3H). ^13^C NMR (126 MHz, CDCl_3_) *δ* 159.9, 145.0, 141.9, 141.6, 136.8, 135.3, 134.1, 133.9, 130.4, 127.9, 126.8, 126.8, 124.6, 114.2, 99.6, 98.6, 55.4, 30.0, 29.9. HRMS (ESI) *m*/*z*: [M + H]^+^ calculated for C_21_H_19_N_4_OS, 375.1274; found 375.1263.

#### 4-(1,5-Dimethyl-6-(thiophen-2-yl)-1,5-dihyrodipyrrolo[3,2-*b*:3′,2′-*e*]pyrazin-2-yl)benzonitrile (4d)

Light brown powder. ^1^H NMR (500 MHz, CDCl_3_) *δ* 7.80 (d, *J* = 8.2 Hz, 2H), 7.73 (d, *J* = 8.3 Hz, 2H), 7.49 (d, *J* = 5.1 Hz, 1H), 7.43–7.40 (m, 1H), 7.23–7.19 (m, 1H), 6.87 (s, 1H), 6.84 (s, 1H), 4.08 (s, 3H), 3.96 (s, 3H). ^13^C NMR (126 MHz, CDCl_3_) *δ* 142.3, 142.0, 142.0, 138.4, 136.6, 135.5, 134.4, 133.5, 132.5, 129.3, 128.0, 127.2, 127.2, 118.6, 111.8, 101.1, 99.5, 30.3, 30.0. HRMS (ESI) *m*/*z*: [M + H]^+^ calculated for C_21_H_16_N_5_S, 370.1121; found 370.1132.

#### 1,5-Dimethyl-2-(4-nitrophenyl)-6-(thiophen-2-yl)-1,5-dihyro dipyrrolo[3,2-*b*:3′,2′-*e*]pyrazine (4e)

HRMS (ESI) *m*/*z*: [M + H]^+^ calculated for C_20_H_16_N_5_O_2_S, 390.1019; found 390.1028.

#### 1,5-Dimethyl-2-(thiophen-2-yl)-6-(4-(trifluoromethyl)phenyl)-1,5-dihyrodipyrrolo[3,2-*b*:3′,2′-*e*]pyrazine (7f)

Light brown solid. ^1^H NMR (500 MHz, CDCl_3_) *δ* 7.78 (d, *J* = 8.3 Hz, 2H), 7.74 (d, *J* = 8.3 Hz, 2H), 7.48 (dd, *J* = 5.1, 0.8 Hz, 1H), 7.42–7.39 (m, 1H), 7.20 (dd, *J* = 5.1, 3.7 Hz, 1H), 6.88 (s, 1H), 6.81 (s, 1H), 4.08 (s, 3H), 3.96 (s, 3H). ^13^C NMR (126 MHz, CDCl_3_) *δ* 142.8, 142.1, 141.9, 138.0, 135.7, 135.1, 134.6, 133.70, 130.5, 129.2, 128.0, 127.1, 125.7 (q, *J* = 3.6 Hz), 125.1, 122.9, 100.5, 99.5, 30.2, 30.0. HRMS (ESI) *m*/*z*: [M + H]^+^ calculated for C_21_H_16_F_3_N_4_S, 413.1024; found 413.1035.

#### 1,5-Dimethyl-2-(thiophen-3-yl)-6-(4-(trifluoromethyl)phenyl)-1,5-dihyrodipyrrolo[3,2-*b*:3′,2′-*e*] pyrazine (7g)

Pale yellow crystals. ^1^H NMR (500 MHz, CDCl_3_) *δ* 7.78 (d, *J* = 8.3 Hz, 2H), 7.74 (d, *J* = 8.2 Hz, 2H), 7.59 (d, *J* = 1.7 Hz, 1H), 7.50 (dd, *J* = 4.8, 3.0 Hz, 1H), 7.43 (d, *J* = 4.8 Hz, 1H), 6.82 (s, 1H), 6.80 (s, 1H), 4.03 (s, 3H), 3.97 (s, 3H). ^13^C NMR (126 MHz, CDCl_3_) *δ* 142.4, 142.0, 141.7, 140.1, 135.7, 135.3, 134.4, 132.7, 130.4, 129.2, 128.0, 126.4, 125.7 (q, *J* = 3.7 Hz), 125.1, 124.0, 100.4, 98.8, 30.0, 30.2. HRMS (ESI) *m*/*z*: [M + H]^+^ calculated for C_21_H_16_F_3_N_4_S, 413.1042; found 413.1031.

## 5. Conclusions

We have demonstrated the design, synthesis, and characterization of DPP-based chromophores with donor–acceptor molecular architecture with well established and straightforward methodologies. The structural, optoelectronic, and thermal properties were investigated in detail. X-ray crystallographic analysis of 7g, reveals π-conjugated framework with a planar geometry. The abilities of these chromophores to function as pH sensors were demonstrated with dramatic color changes upon the introduction of acid. These findings suggest that suitable design of the molecules and a sound understanding of their spectroscopic properties could enable to develop promising pH sensors. Surface morphological studies reveal the formation of microrods with diverse particle size. Undoubtedly, our results provide important guidelines for designing DPP-based molecular semiconductors, indicating that through rational design and synthesis, DPP can be a highly favorable building block for efficient electron charge-transport in optoelectronics.

## Conflicts of interest

There are no conflicts to declare.

## Supplementary Material

RA-008-C7RA12527E-s001

RA-008-C7RA12527E-s002
