# Supplementary material for: Unveiling the photophysical and morphological properties of an acidochromic thiophene flanked dipyrrolopyrazine-based chromophore for optoelectronic application
Source: RSC Adv. 2018 Jan 9;8(4):2004–14. doi: 10.1039/c7ra12527e (PMC9077264; doi:10.1039/c7ra12527e)
Supplement: RA-008-C7RA12527E-s001 [file RA-008-C7RA12527E-s001.pdf]

Electronic Supplementary Material (ESI) for RSC Advances.
This journal is © The Royal Society of Chemistry 2017

Supporting Information

Unveiling the photophysical and morphological properties of Acidochromic thiophene flanked Dipyrolopyrazine-based chromophore for optoelectronic application

Puttavva Meti and Young-Dae Gong*

Innovative Drug Library Research Center, Department of Chemistry, College of Science, Dongguk University, 26, 3-ga, Pil-dong, Jung-gu, Seoul 04620, Korea

Corresponding author's E-Mail: ydgong@dongguk.edu

Contents

1. ^{1}H NMR ^{13}C NMR of chromophores **3(d-e)** ……………………………………S2-S3

2. ^{1}H NMR ^{13}C NMR of chromophores **6g** ……………………………….. …....S4

3. ^{1}H NMR ^{13}C NMR of chromophores **4(a-d)** ……………………………………..S5-S8

4. ^{1}H NMR ^{13}C NMR of chromophores **7(f-g)** ……………………………………...S9-S10

5. HRMS spectra of chromophores **3(a-e)** ………………………………………….S11-S12

6. HRMS spectra of chromophores **6(f-g)** ………………………………………….S13

7. HRMS spectra of chromophores **4(a-e)** …………………………………………S14

8. HRMS spectra of chromophores **7(f-g)** …………………………………………S15

9. Absorption and emission spectra of 4a in different solvents ……………………S16

10. Photographic images of chromophores …………………………………… S17

11. Single crystal XRD data of **7g**……………………………………………… S17-S26

^{1}H NMR ^{13}C NMR of compounds

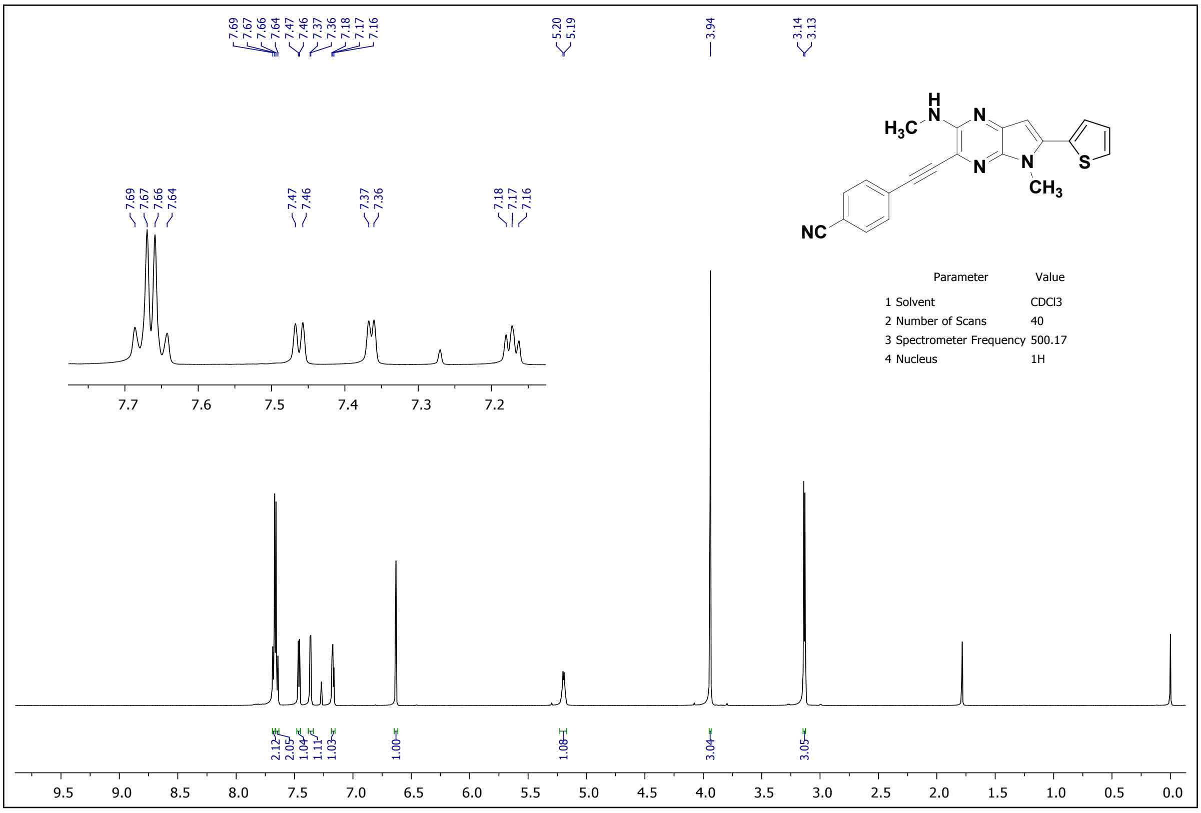

^{1}H NMR – 3d

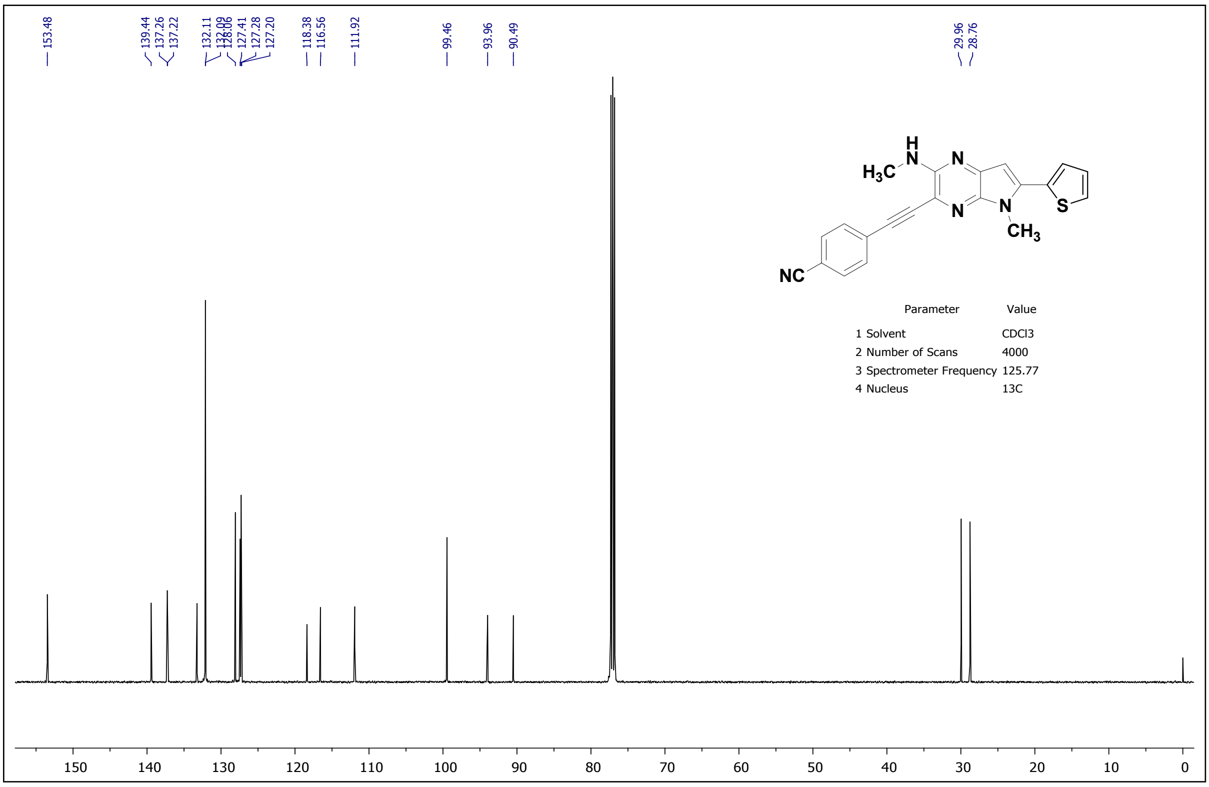

^{13}C NMR – 3d

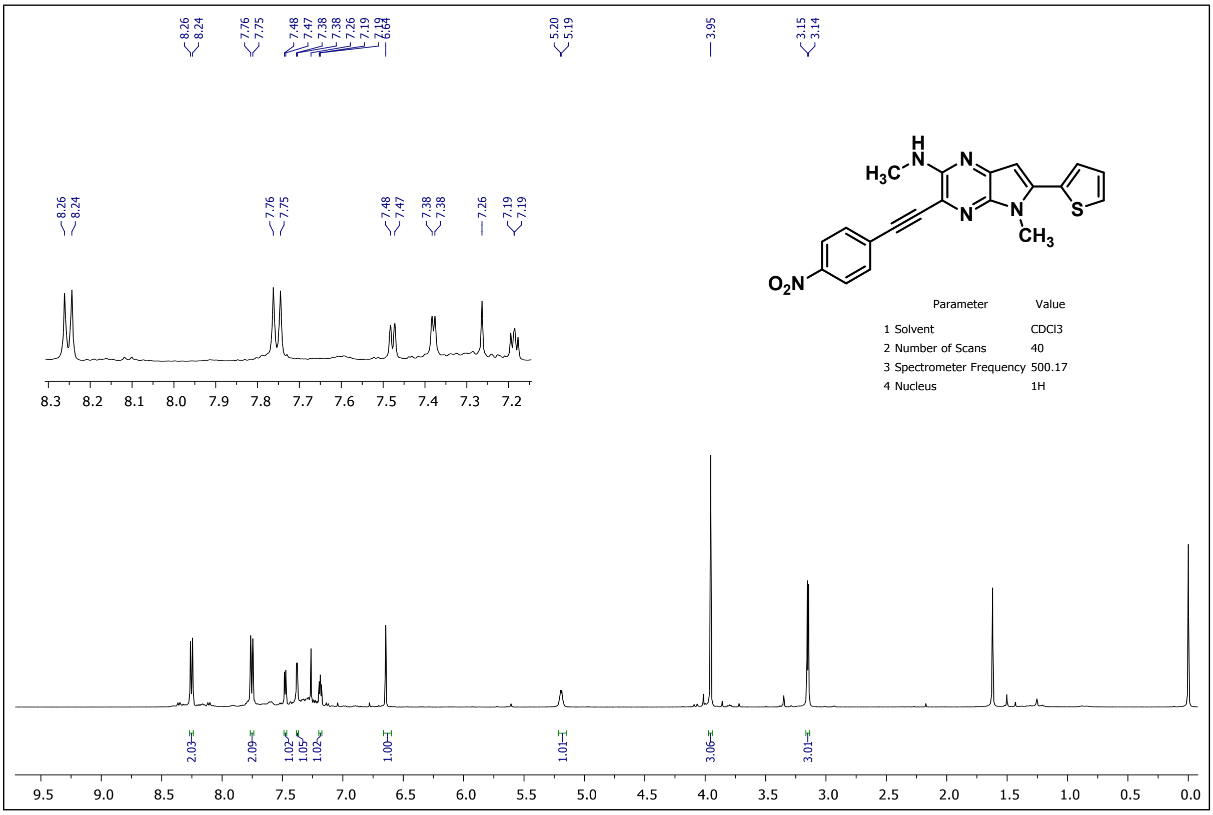

^{1}H NMR – 3e

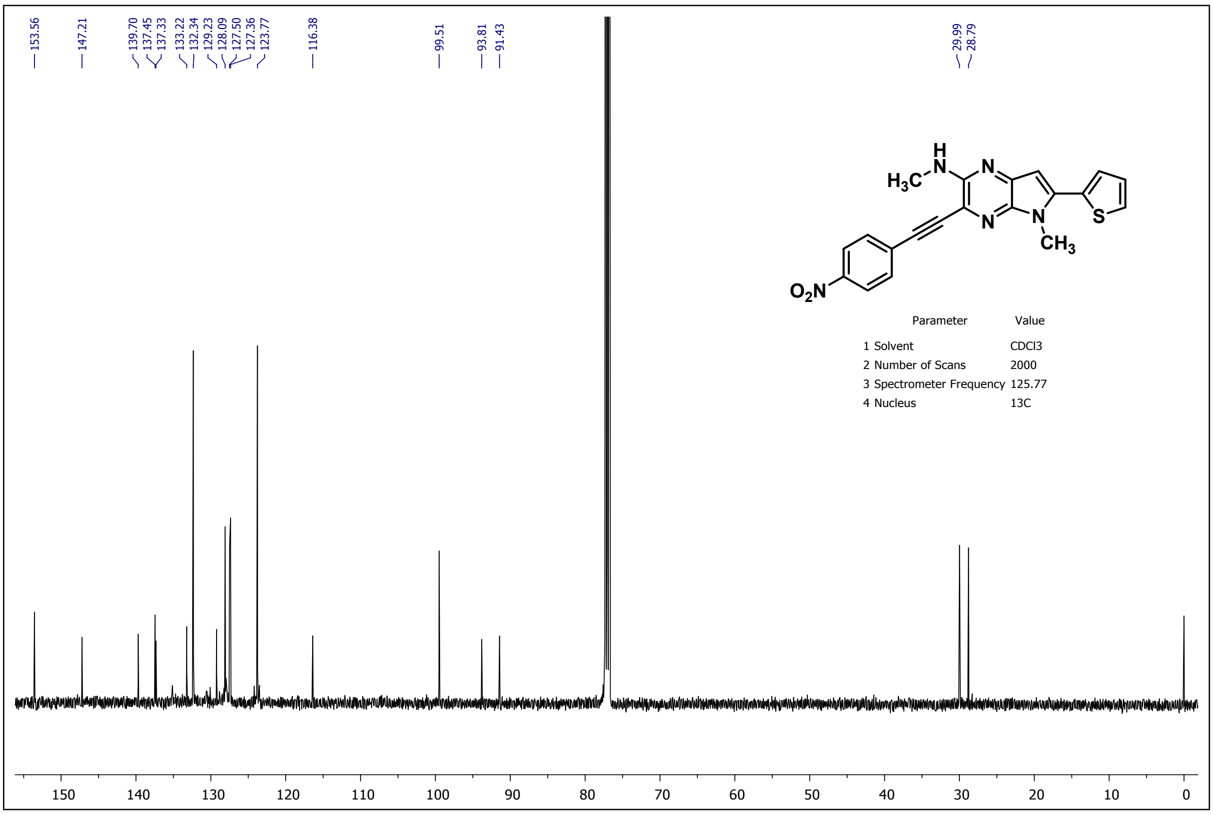

^{13}C NMR – 3e

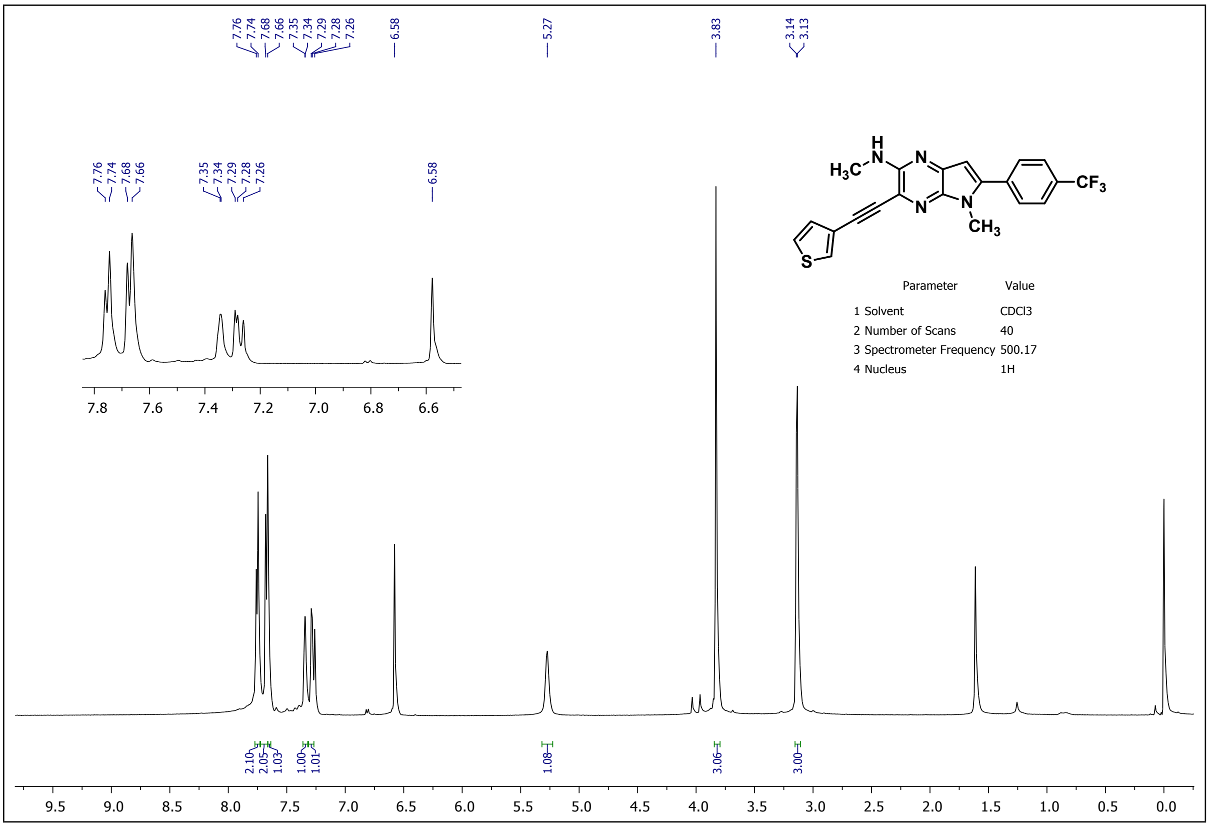

^{1}H NMR – 6g

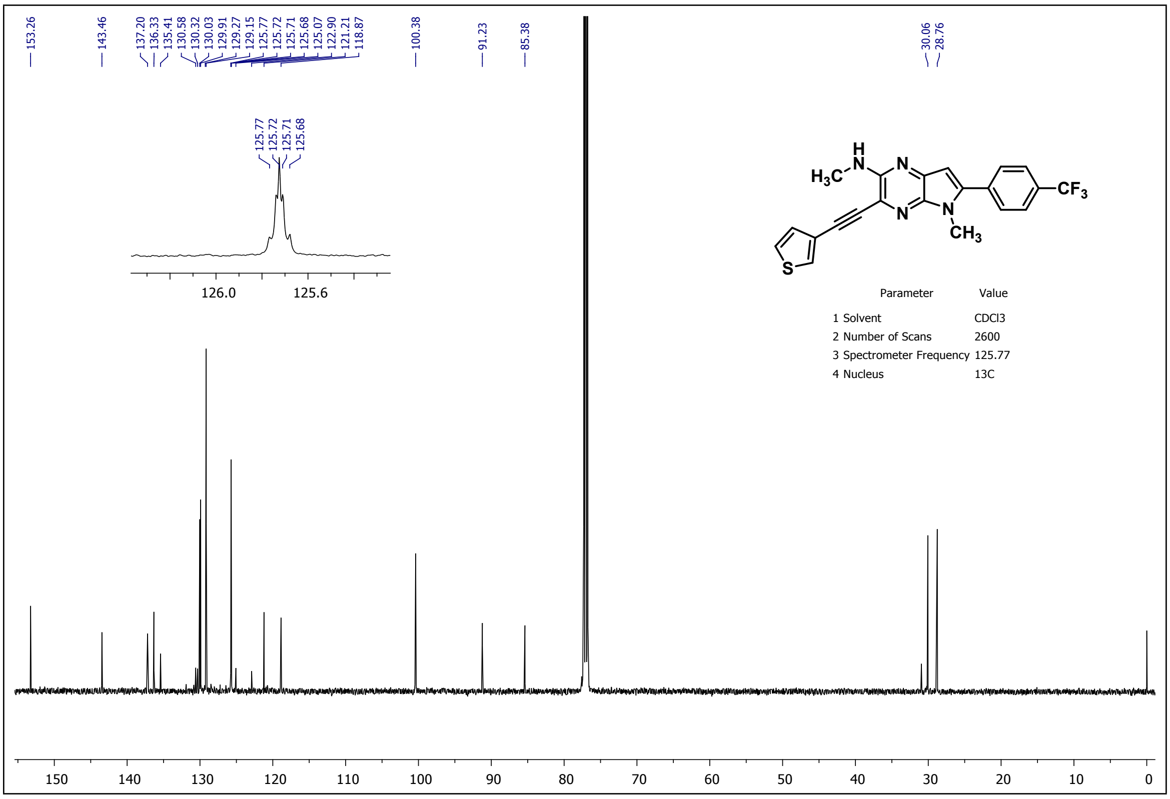

^{13}C NMR – 6g

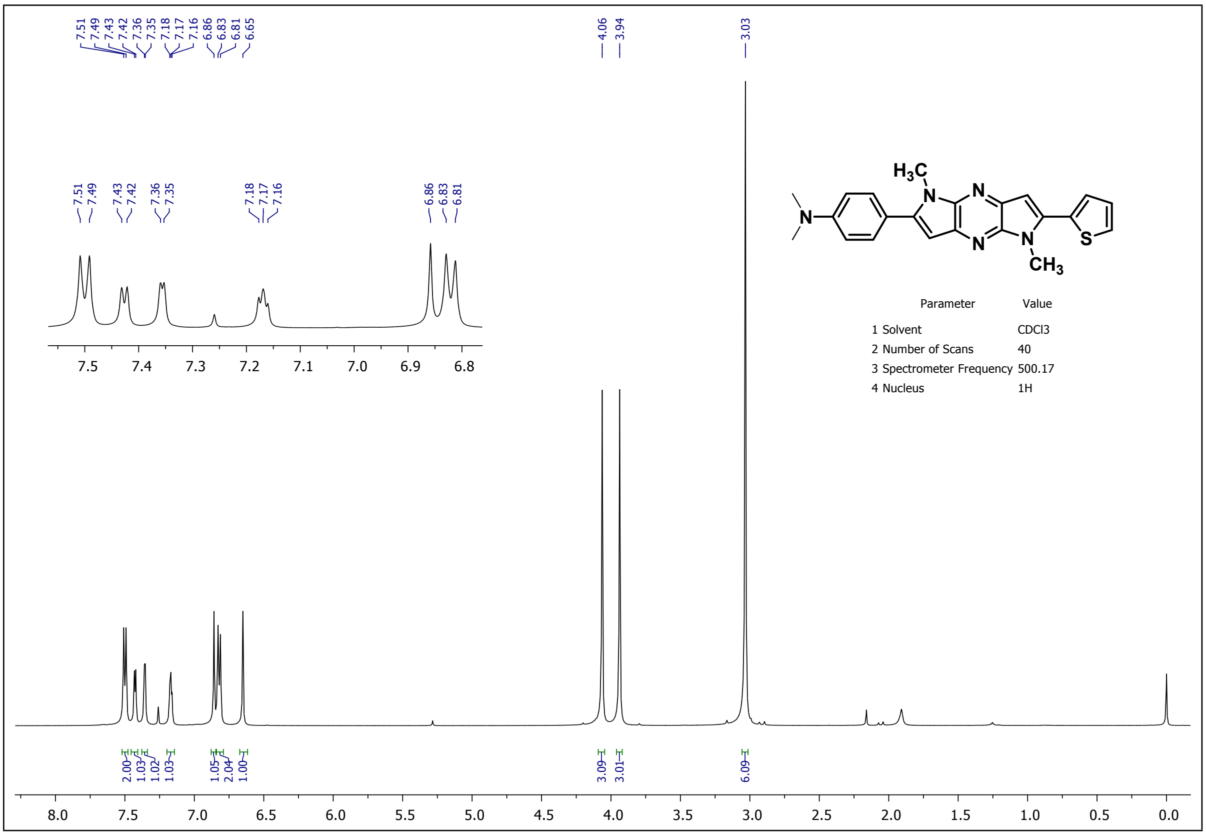

^{1}H NMR – 4a

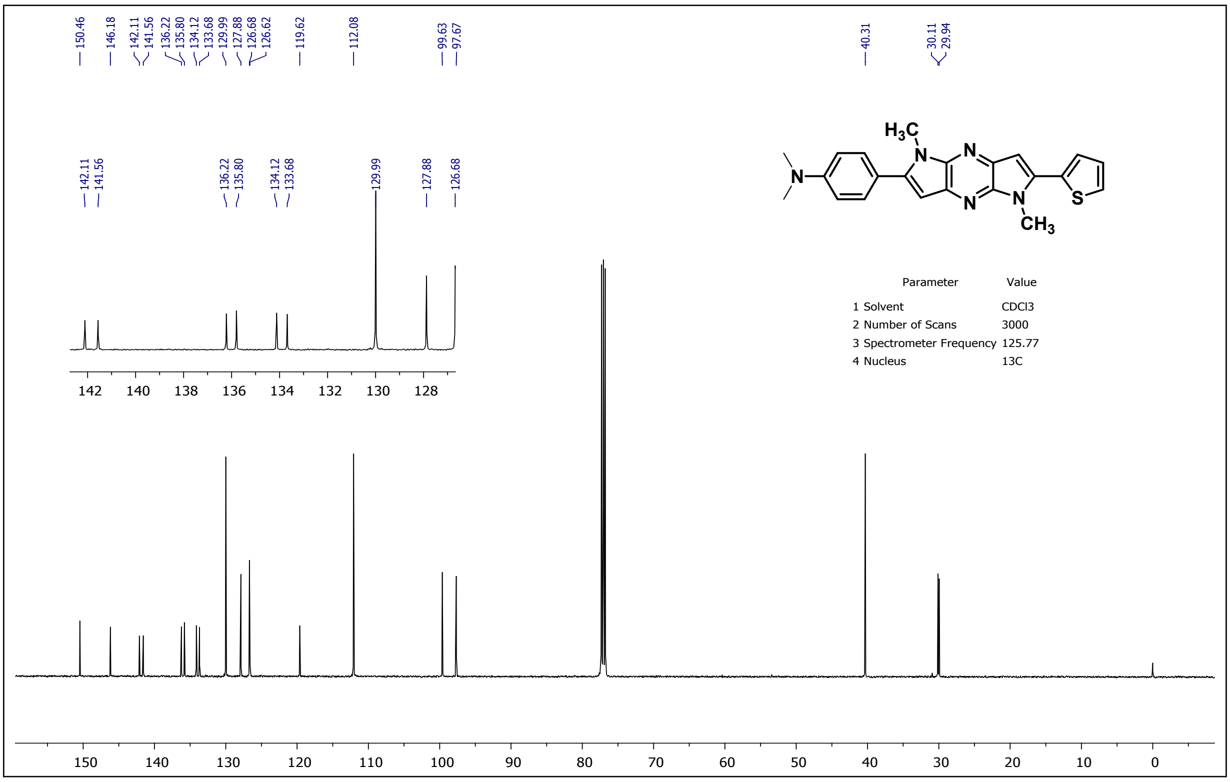

^{13}C NMR – 4a

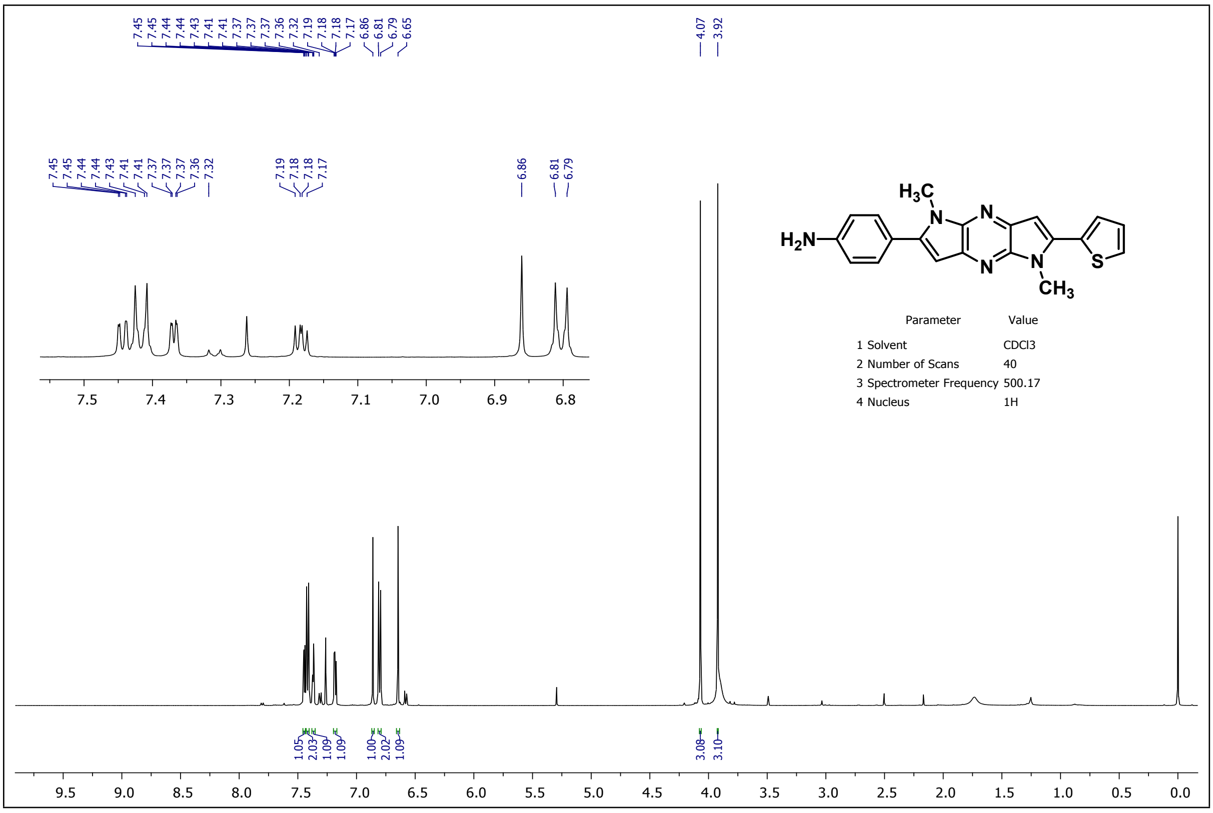

^{1}H NMR – 4b

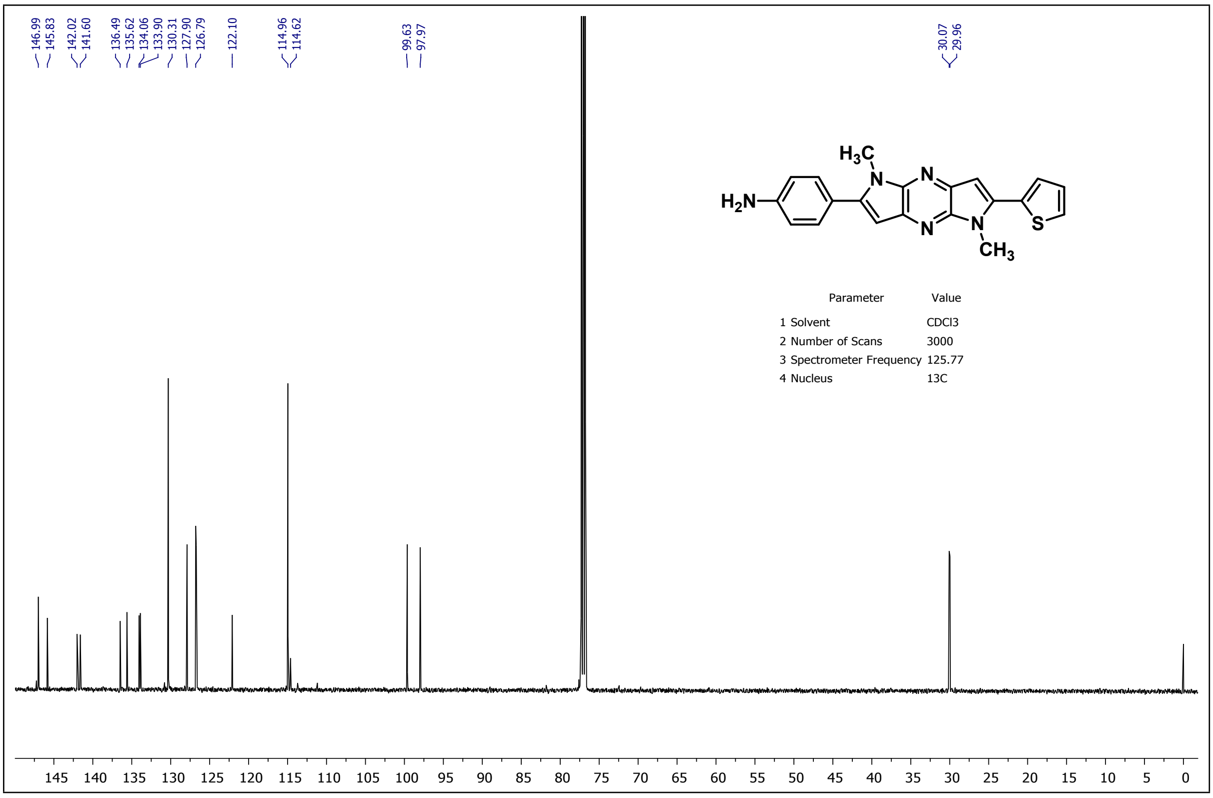

^{13}C NMR – 4b

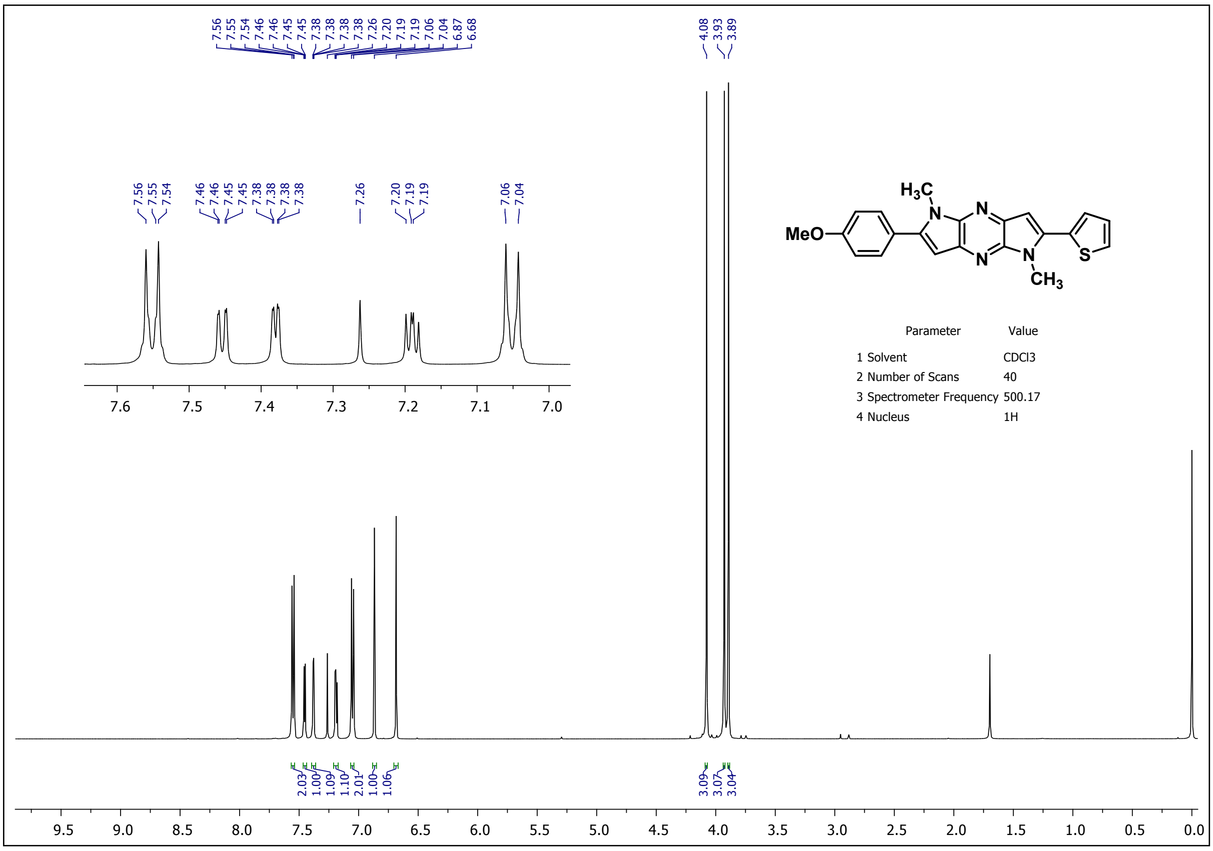

^{1}H NMR – 4c

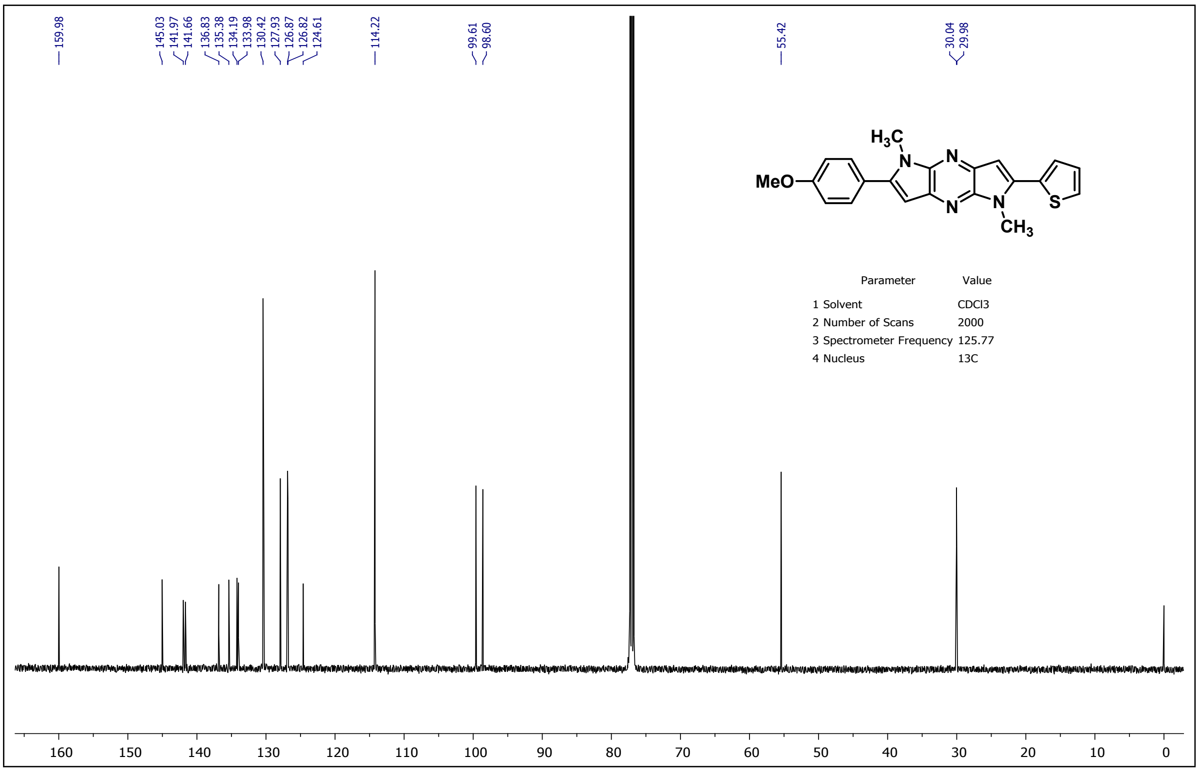

^{13}C NMR – 4c

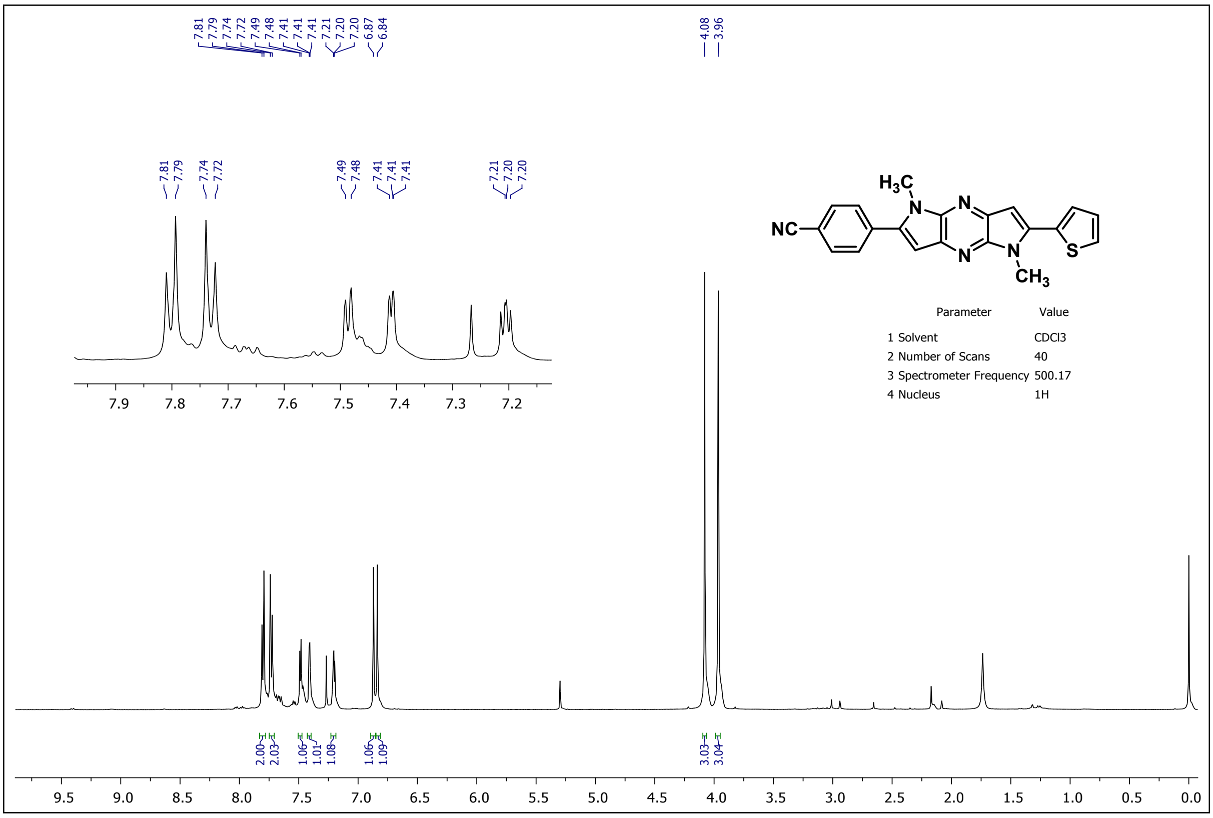

^{1}H NMR – 4d

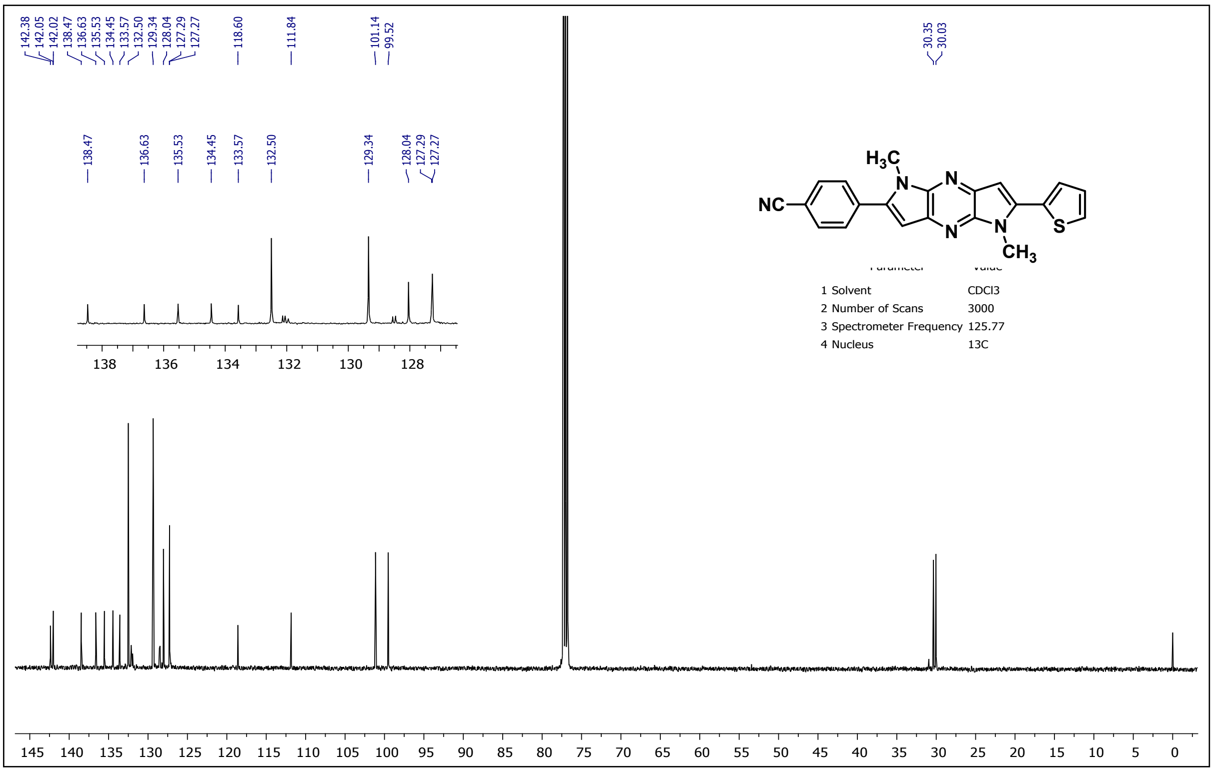

^{13}C NMR – 4d

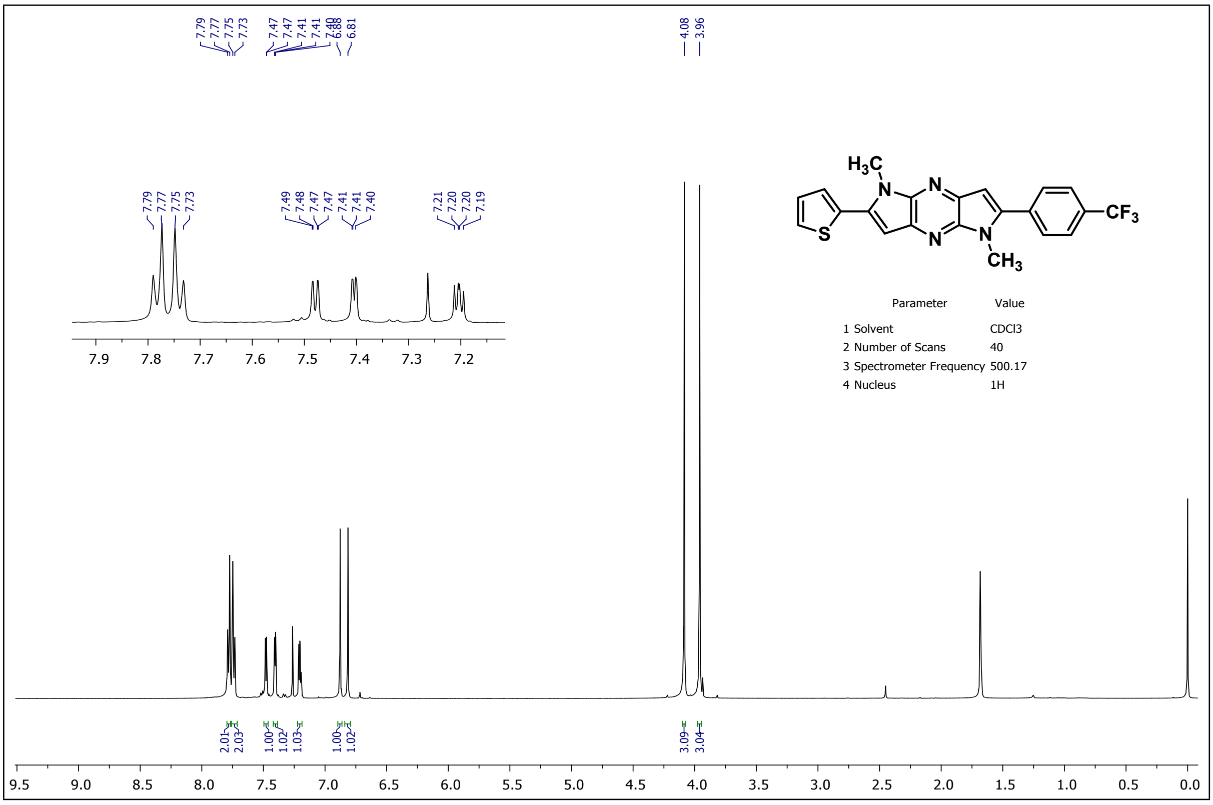

^{1}H NMR – 7f

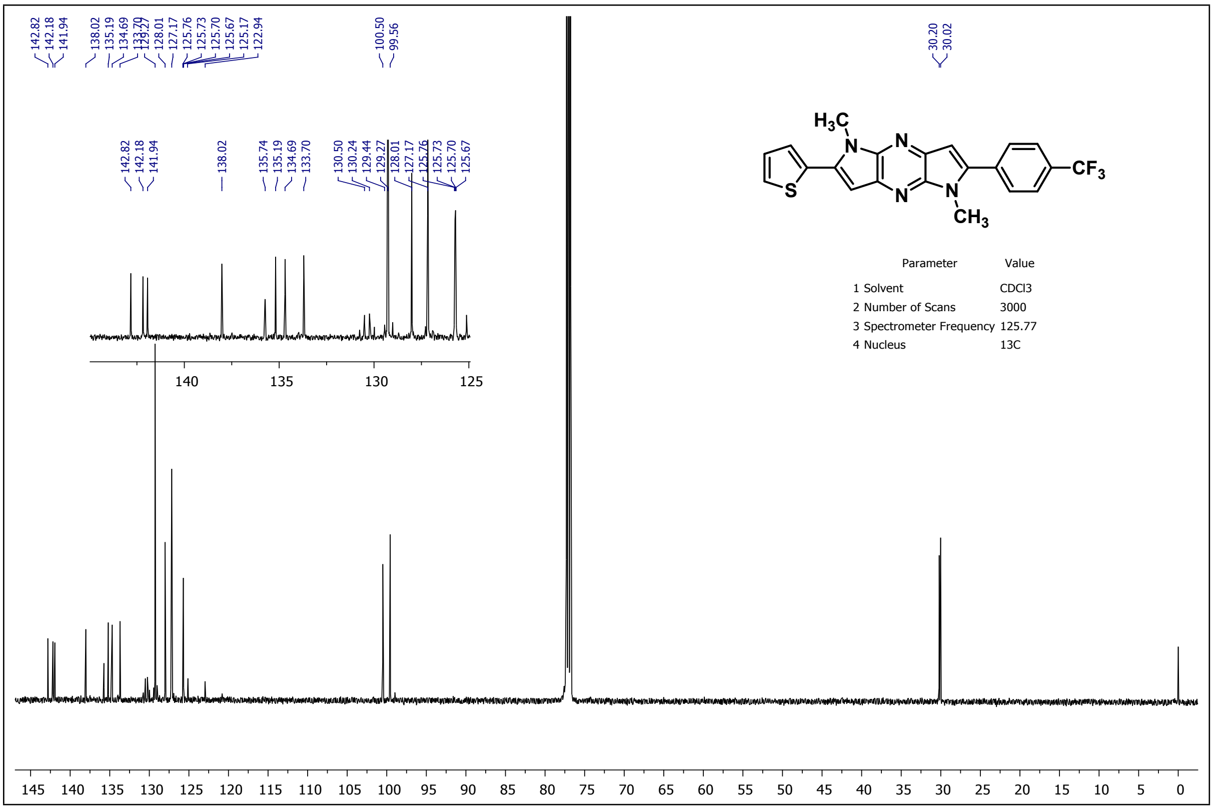

^{13}C NMR – 7f

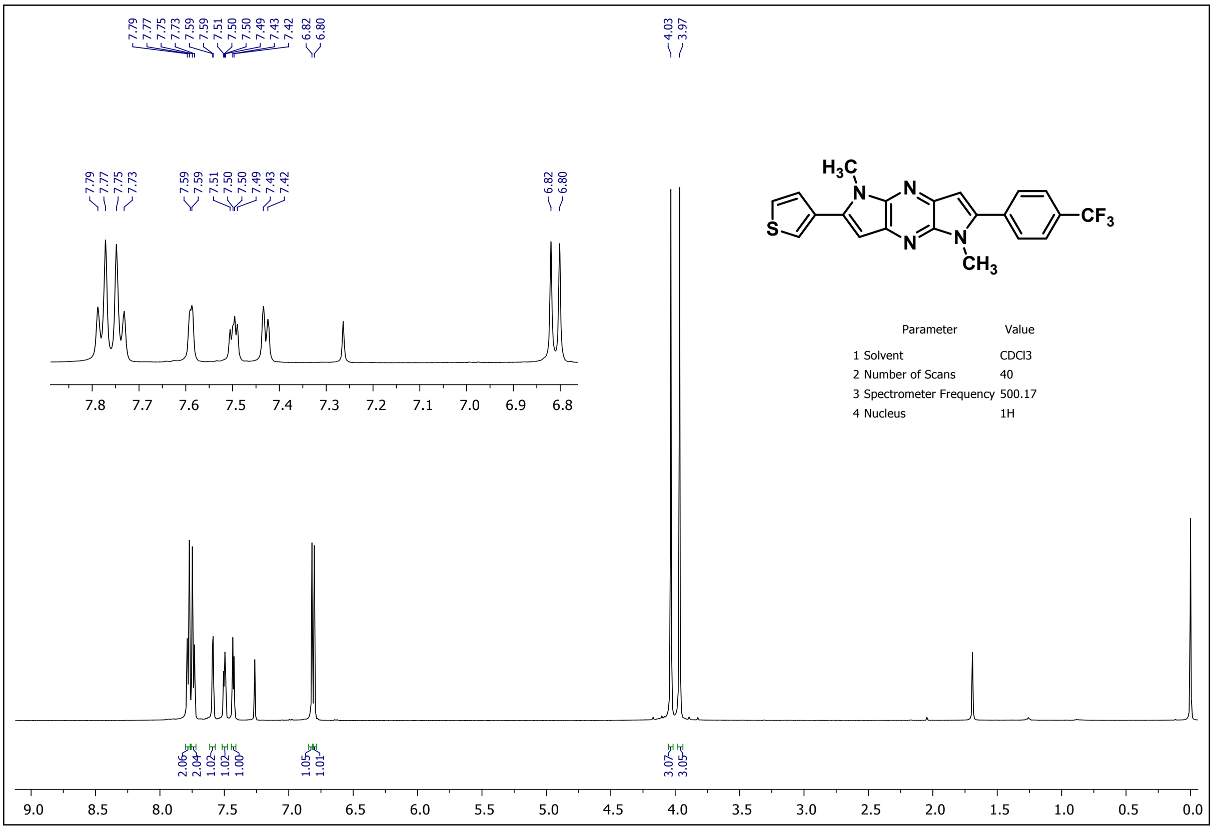

^{1}H NMR – 7g

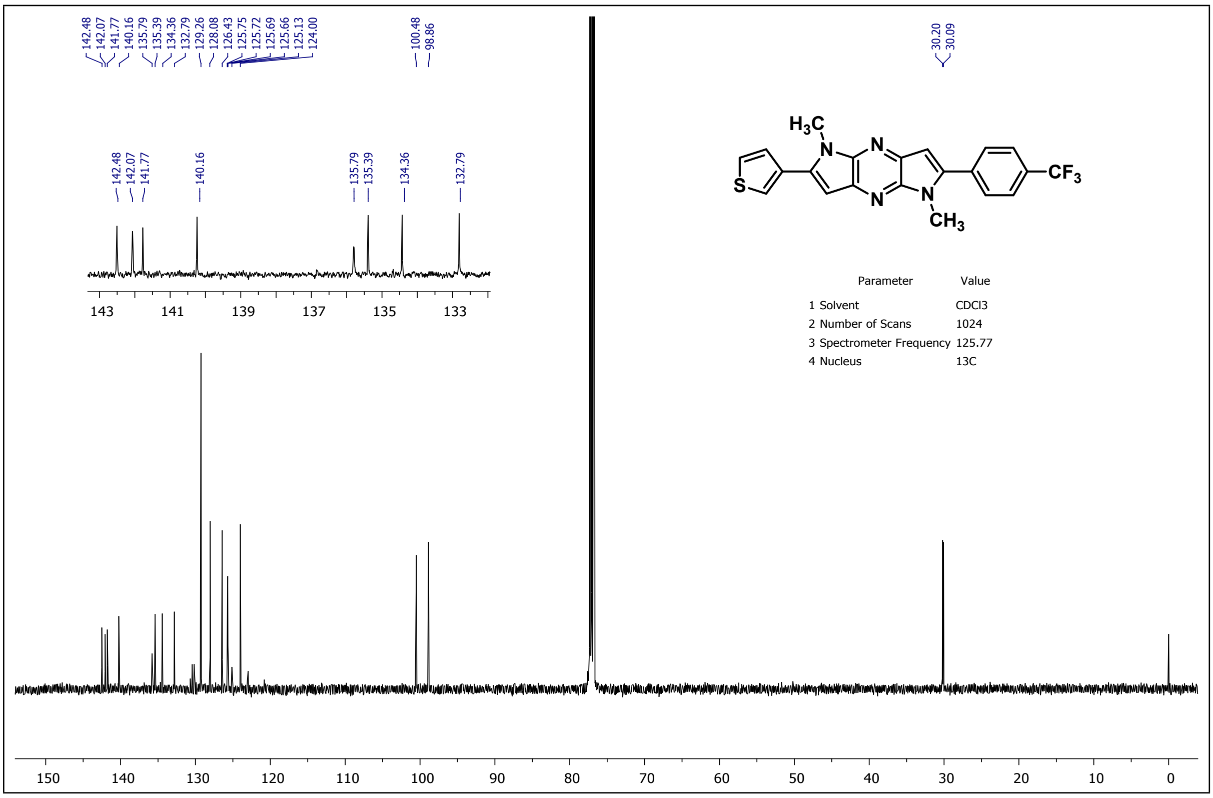

^{13}C NMR – 7g

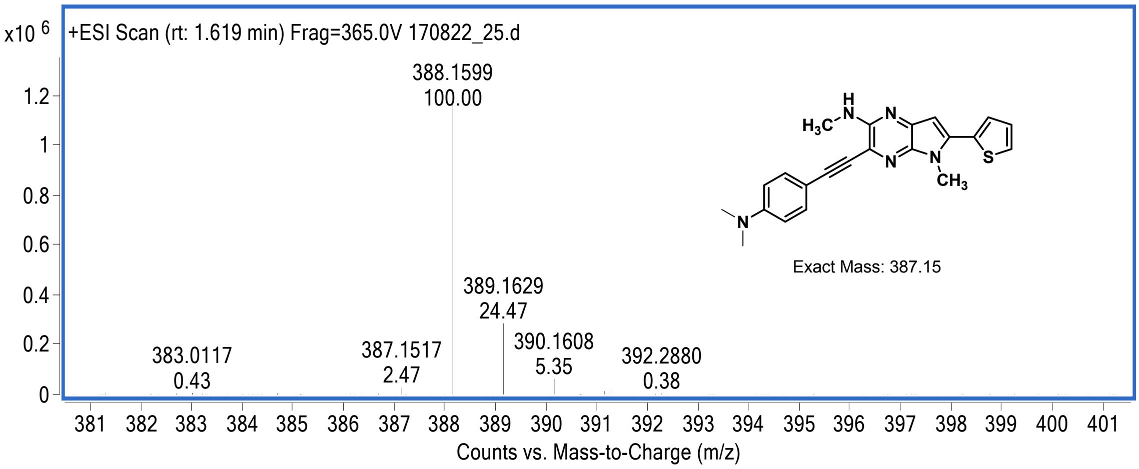

HRMS – 3a

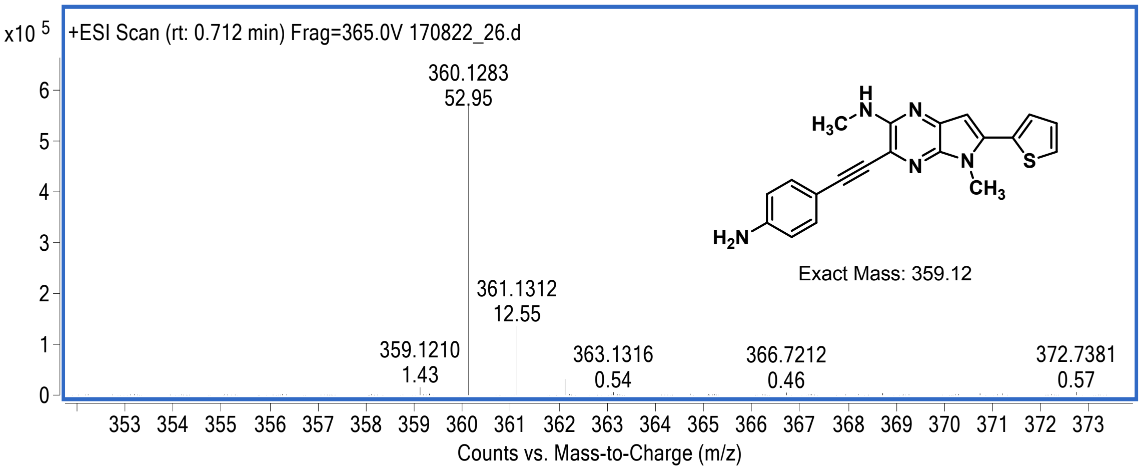

HRMS – 3b

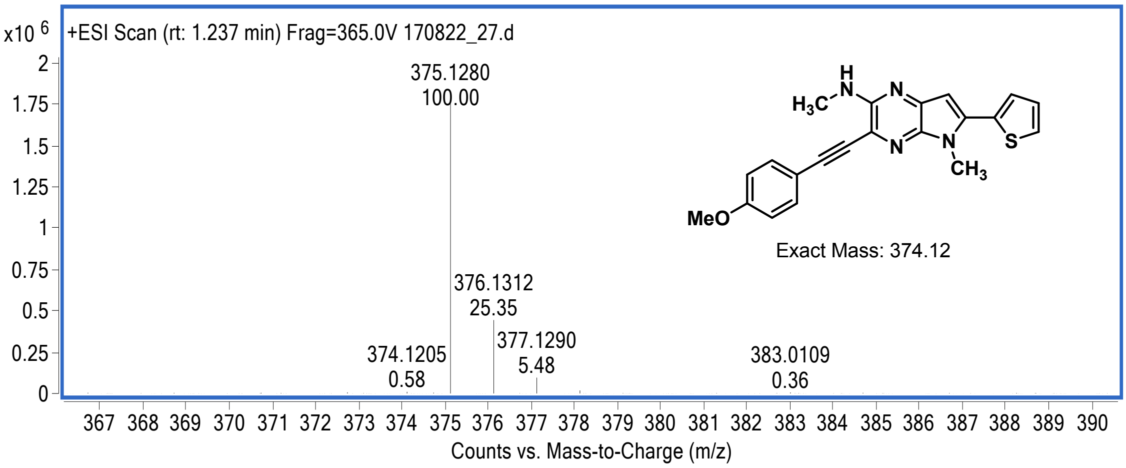

HRMS – 3c

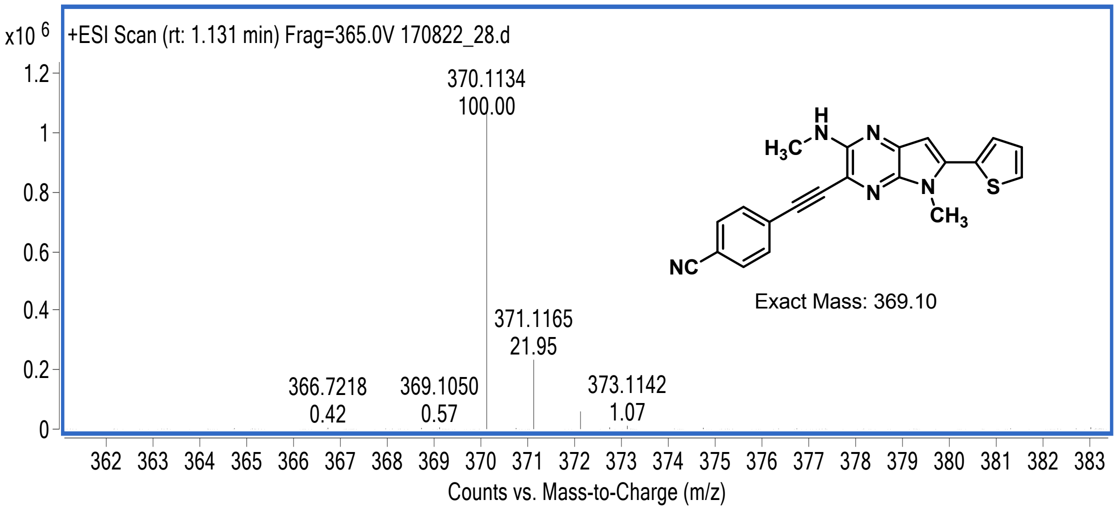

HRMS – 3d

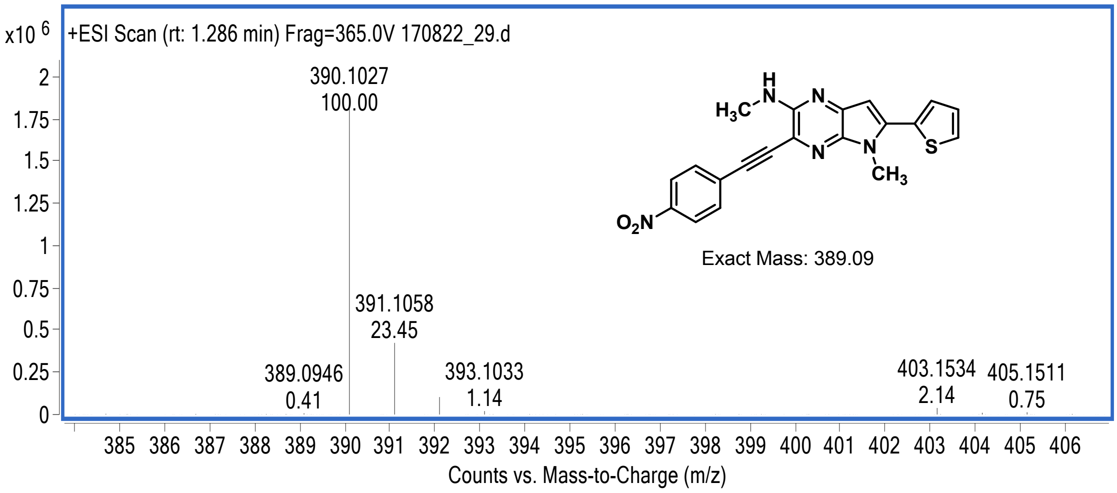

HRMS – 3e

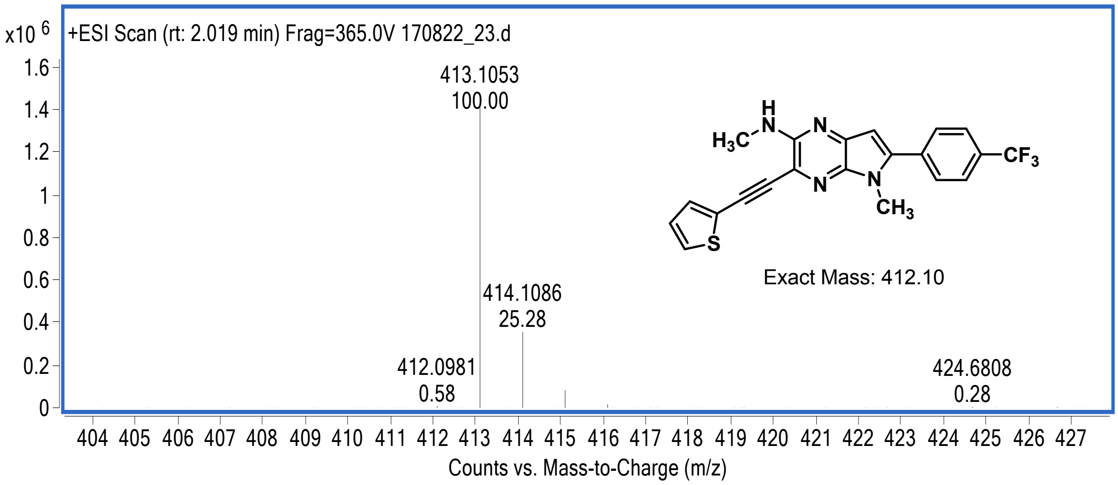

HRMS – 6f

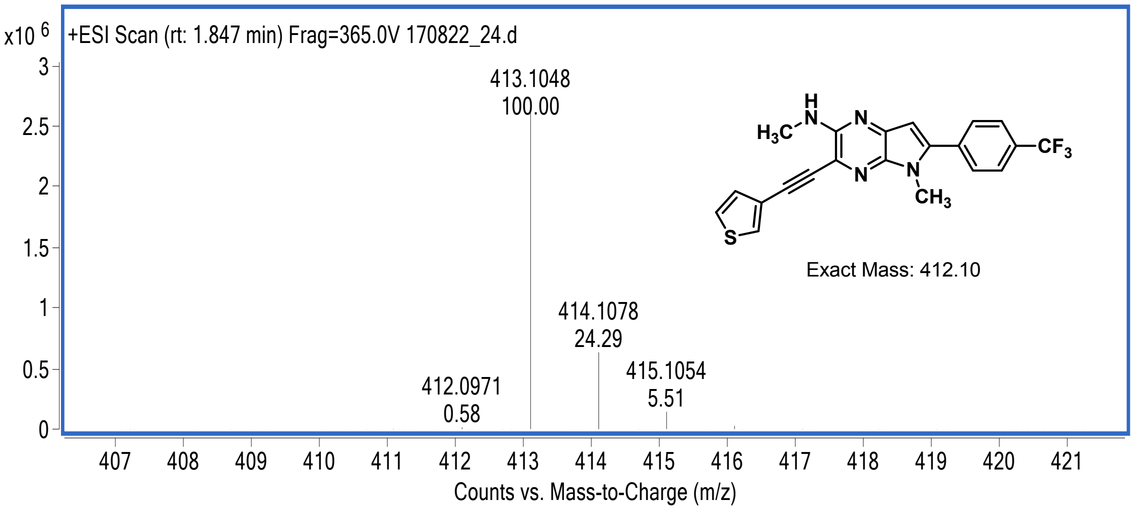

HRMS – 6g

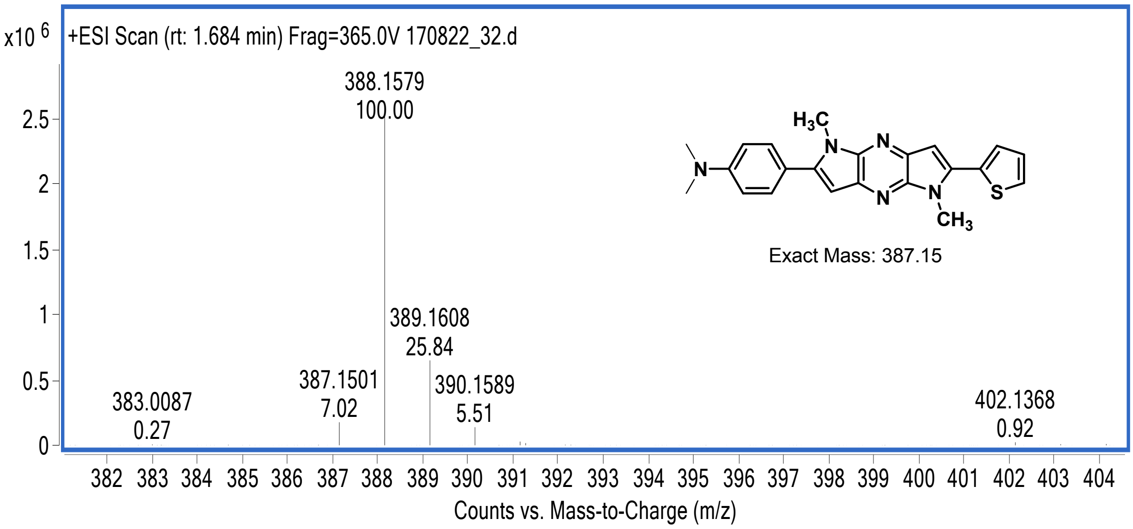

HRMS – 4a

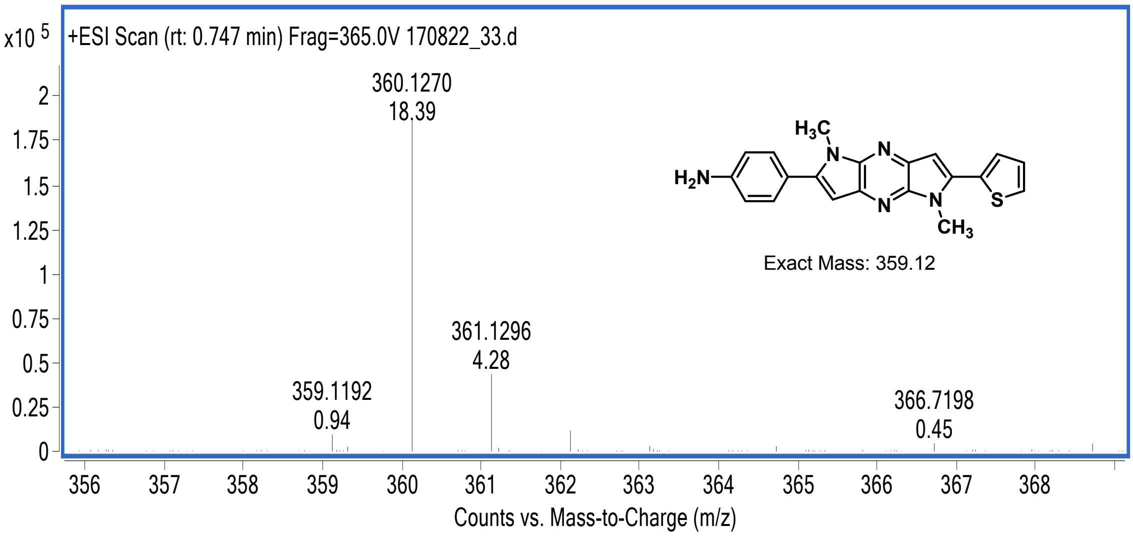

HRMS – 4b

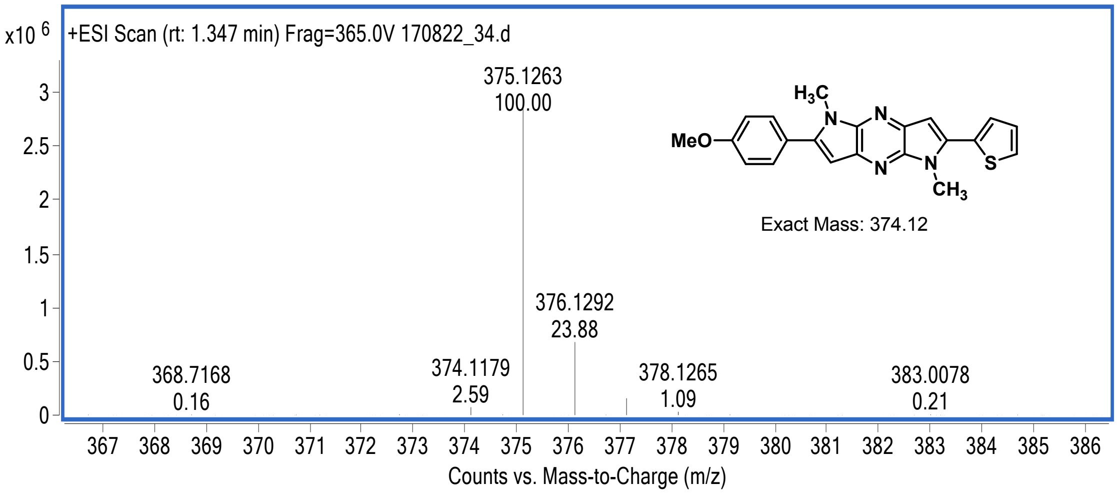

HRMS – 4c

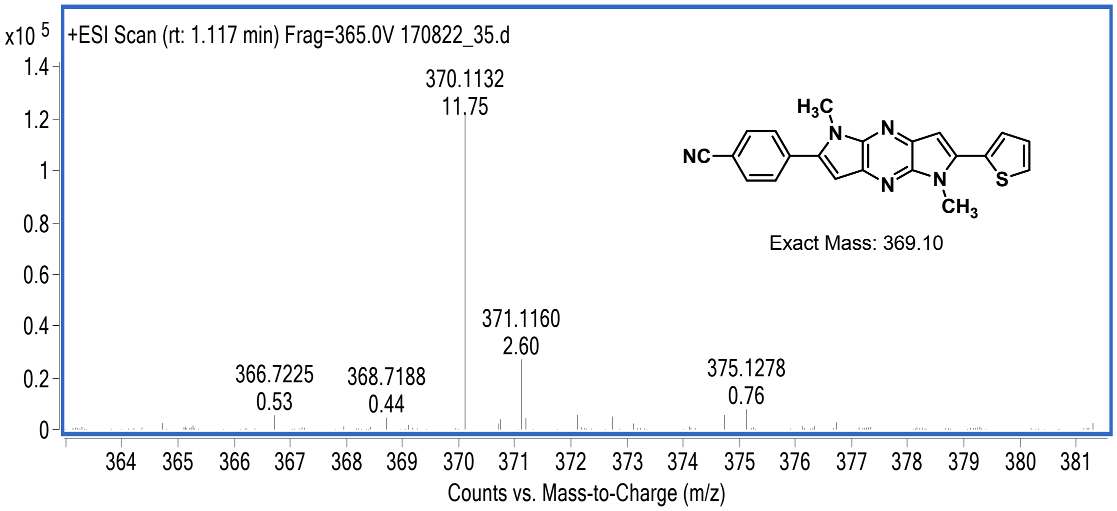

HRMS – 4d

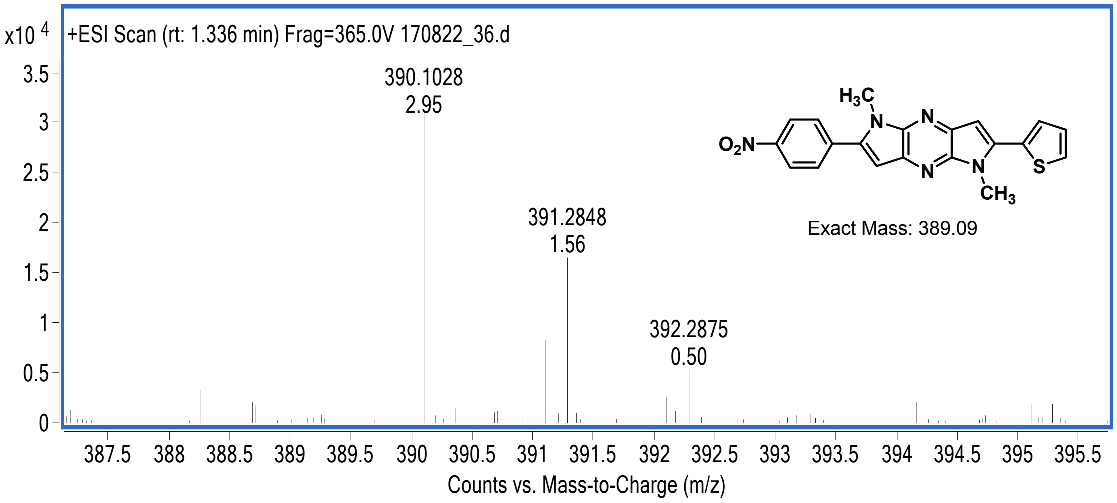

HRMS – 4e

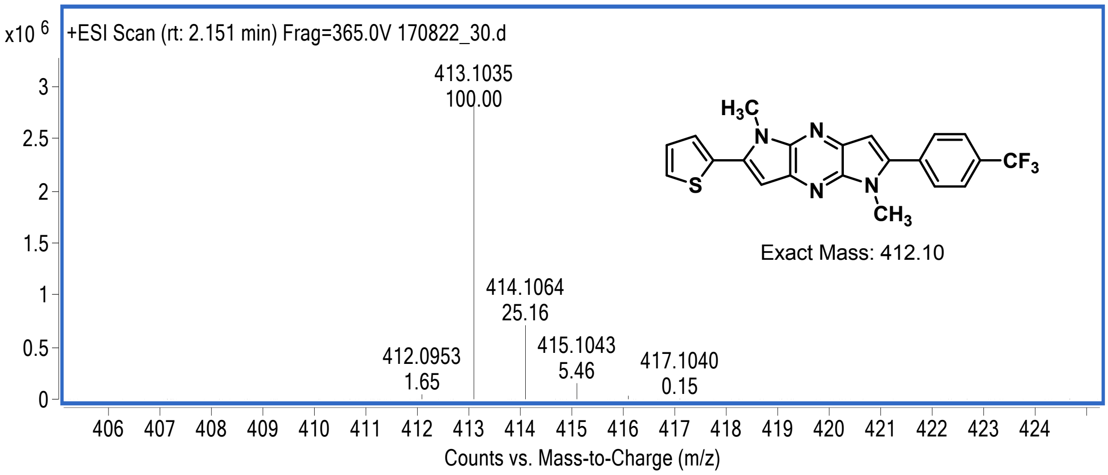

HRMS – 7f

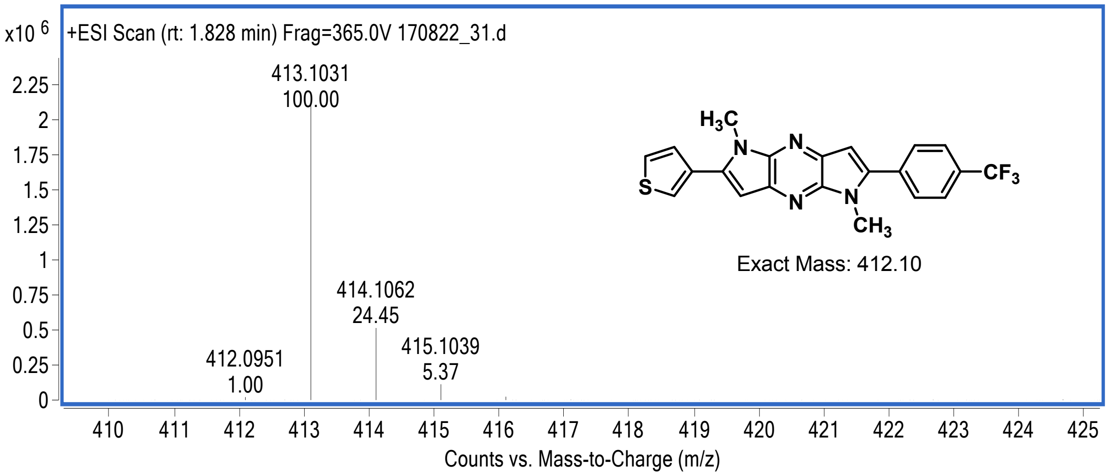

HRMS – 7g

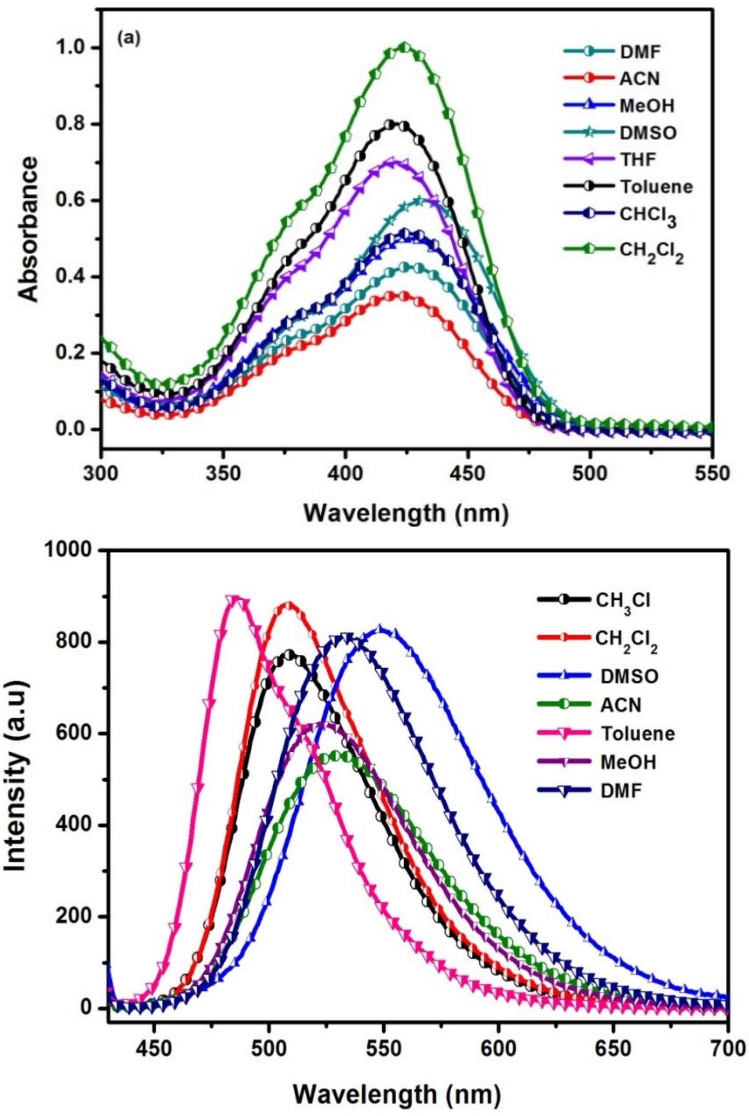

Fig. 1 a) Absorption b) emission spectra of **4a** in solvents of varying polarity and proticity.

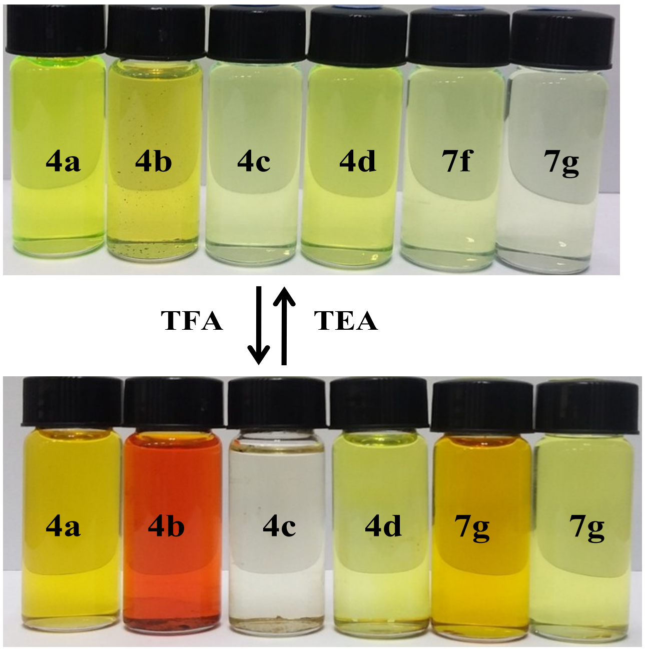

Fig. 2 Photographic images of chromophores showing color change before and after addition of TFA under normal light.

Table 1.Crystal data and structure refinement for chromophores **7g**.

Identification code	20170818_0m	
Empirical formula	C21 H15 F3 N4 S	
Formula weight	412.43	
Temperature	296(1) K	
Wavelength	0.71073 Å	
Crystal system	Monoclinic	
Space group	P2 (1)/c	
Unit cell dimensions	a = 15.0769(2) Å	α= 90°.
	b = 8.80100(10) Å	β= 115.2030(10)° .
	c = 15.4166(2) Å	γ = 90°.
Volume	1850.92(4) $Å^3$	
Z	4	
Density (calculated)	1.480 Mg/m^3	
Absorption coefficient	0.220 mm^{-1}	
F(000)	848	
Crystal size	0.38 x 0.22 x 0.04 mm^3	
Theta range for data collection	1.49 to 28.28°	
Index ranges	-20<=h<=18, 0<=k<=11, 0<=l<=20	

Reflections collected	4596
Independent reflections	4596 [R(int) = 0.0000]
Completeness to theta = 28.28°	99.8 %
Absorption correction	Multi-scan
Max. and min. transmission	0.9913 and 0.9212
Refinement method	Full-matrix least-squares on F^2
Data / restraints / parameters	4596 / 0 / 262
Goodness-of-fit on F^2	1.554
Final R indices [I>2sigma(I)]	R1 = 0.1120, wR2 = 0.3494
R indices (all data)	R1 = 0.1359, wR2 = 0.3831
Largest diff. peak and hole	1.296 and -0.778 e.Å^{-3}

Table 2. Atomic coordinates (x 10^4) and equivalent isotropic displacement parameters (Å^{2}x 10^3) For **7g**. U(eq) is defined as one third of the trace of the orthogonalized U^{ij} tensor.

	x	y	z	U(eq)
S(1)	12797(1)	242(2)	1551(1)	69(1)
F(1)	5503(3)	3146(10)	7117(3)	200(3)
F(2)	4561(4)	2178(11)	5992(6)	245(5)
F(3)	4815(8)	4247(10)	5947(8)	321(7)
N(1)	11301(2)	2151(3)	3692(2)	36(1)
N(2)	10332(2)	2756(3)	4542(2)	39(1)
N(3)	9138(2)	216(3)	3419(2)	37(1)
N(4)	8150(2)	819(3)	4255(2)	40(1)
C(1)	11791(3)	441(5)	1746(3)	52(1)
C(2)	11993(2)	933(4)	2650(2)	39(1)
C(3)	13013(3)	1165(5)	3193(3)	57(1)
C(4)	13583(3)	832(5)	2652(3)	49(1)
C(5)	11249(2)	1087(4)	3017(2)	38(1)
C(6)	10423(2)	174(4)	2795(2)	41(1)
C(7)	9959(2)	683(4)	3363(2)	37(1)
C(8)	10519(2)	1936(4)	3919(2)	34(1)
C(9)	11930(3)	3491(4)	3984(3)	48(1)
C(10)	8952(2)	1023(4)	4053(2)	35(1)
C(11)	9517(2)	2237(4)	4614(2)	37(1)
C(12)	9050(3)	2763(4)	5188(2)	42(1)
C(13)	8221(2)	1902(4)	4950(2)	39(1)

C(14)	7535(3)	-497(5)	3952(3)	53(1)
C(15)	7449(2)	2114(4)	5281(2)	39(1)
C(16)	7713(3)	2598(4)	6227(2)	42(1)
C(17)	6995(3)	2912(4)	6537(3)	47(1)
C(18)	6020(3)	2777(5)	5933(3)	53(1)
C(19)	5745(3)	2284(6)	4995(3)	64(1)
C(20)	6466(3)	1977(5)	4680(3)	57(1)
C(21)	5262(3)	3110(7)	6261(4)	72(1)

Table 3.Bond lengths [Å] and angles [°] for **7g**.

S(1)-C(1)	1.676(4)
S(1)-C(4)	1.686(4)
F(1)-C(21)	1.211(6)
F(2)-C(21)	1.260(8)
F(3)-C(21)	1.188(7)
N(1)-C(8)	1.377(4)
N(1)-C(5)	1.377(4)
N(1)-C(9)	1.459(4)
N(2)-C(8)	1.324(4)
N(2)-C(11)	1.360(4)
N(3)-C(10)	1.331(4)
N(3)-C(7)	1.341(4)
N(4)-C(10)	1.383(4)
N(4)-C(13)	1.403(4)
N(4)-C(14)	1.433(5)
C(1)-C(2)	1.364(5)
C(1)-H(1)	0.9300
C(2)-C(3)	1.418(5)
C(2)-C(5)	1.463(4)
C(3)-C(4)	1.459(5)
C(3)-H(3)	0.9300
C(4)-H(4)	0.9300
C(5)-C(6)	1.397(5)
C(6)-C(7)	1.407(4)

C(6)-H(6A)	0.9300
C(7)-C(8)	1.430(4)
C(9)-H(9A)	0.9600
C(9)-H(9B)	0.9600
C(9)-H(9C)	0.9600
C(10)-C(11)	1.409(5)
C(11)-C(12)	1.423(4)
C(12)-C(13)	1.371(5)
C(12)-H(12A)	0.9300
C(13)-C(15)	1.469(4)
C(14)-H(14A)	0.9600
C(14)-H(14B)	0.9600
C(14)-H(14C)	0.9600
C(15)-C(20)	1.378(5)
C(15)-C(16)	1.405(5)
C(16)-C(17)	1.386(5)
C(16)-H(16A)	0.9300
C(17)-C(18)	1.370(6)
C(17)-H(17A)	0.9300
C(18)-C(19)	1.392(6)
C(18)-C(21)	1.463(5)
C(19)-C(20)	1.392(5)
C(19)-H(19A)	0.9300
C(20)-H(20A)	0.9300
C(1)-S(1)-C(4)	95.08(18)
C(8)-N(1)-C(5)	108.6(3)
C(8)-N(1)-C(9)	123.2(3)
C(5)-N(1)-C(9)	127.1(3)
C(8)-N(2)-C(11)	110.8(3)
C(10)-N(3)-C(7)	111.9(3)
C(10)-N(4)-C(13)	107.1(3)
C(10)-N(4)-C(14)	122.6(3)
C(13)-N(4)-C(14)	128.8(3)
C(2)-C(1)-S(1)	113.0(3)
C(2)-C(1)-H(1)	123.5
S(1)-C(1)-H(1)	123.5
C(1)-C(2)-C(3)	111.3(3)

C(1)-C(2)-C(5)	123.4(3)
C(3)-C(2)-C(5)	125.1(3)
C(2)-C(3)-C(4)	112.8(3)
C(2)-C(3)-H(3)	123.6
C(4)-C(3)-H(3)	123.6
C(3)-C(4)-S(1)	107.8(3)
C(3)-C(4)-H(4)	126.1
S(1)-C(4)-H(4)	126.1
N(1)-C(5)-C(6)	109.5(3)
N(1)-C(5)-C(2)	123.4(3)
C(6)-C(5)-C(2)	127.0(3)
C(5)-C(6)-C(7)	107.0(3)
C(5)-C(6)-H(6A)	126.5
C(7)-C(6)-H(6A)	126.5
N(3)-C(7)-C(6)	131.3(3)
N(3)-C(7)-C(8)	121.6(3)
C(6)-C(7)-C(8)	107.1(3)
N(2)-C(8)-N(1)	125.3(3)
N(2)-C(8)-C(7)	126.9(3)
N(1)-C(8)-C(7)	107.8(3)
N(1)-C(9)-H(9A)	109.5
N(1)-C(9)-H(9B)	109.5
H(9A)-C(9)-H(9B)	109.5
N(1)-C(9)-H(9C)	109.5
H(9A)-C(9)-H(9C)	109.5
H(9B)-C(9)-H(9C)	109.5
N(3)-C(10)-N(4)	125.0(3)
N(3)-C(10)-C(11)	126.4(3)
N(4)-C(10)-C(11)	108.5(3)
N(2)-C(11)-C(10)	122.4(3)
N(2)-C(11)-C(12)	130.2(3)
C(10)-C(11)-C(12)	107.4(3)
C(13)-C(12)-C(11)	106.9(3)
C(13)-C(12)-H(12A)	126.6
C(11)-C(12)-H(12A)	126.6
C(12)-C(13)-N(4)	110.1(3)
C(12)-C(13)-C(15)	127.2(3)
N(4)-C(13)-C(15)	122.5(3)

N(4)-C(14)-H(14A)	109.5
N(4)-C(14)-H(14B)	109.5
H(14A)-C(14)-H(14B)	109.5
N(4)-C(14)-H(14C)	109.5
H(14A)-C(14)-H(14C)	109.5
H(14B)-C(14)-H(14C)	109.5
C(20)-C(15)-C(16)	118.2(3)
C(20)-C(15)-C(13)	122.8(3)
C(16)-C(15)-C(13)	118.9(3)
C(17)-C(16)-C(15)	120.1(3)
C(17)-C(16)-H(16A)	120.0
C(15)-C(16)-H(16A)	120.0
C(18)-C(17)-C(16)	121.2(3)
C(18)-C(17)-H(17A)	119.4
C(16)-C(17)-H(17A)	119.4
C(17)-C(18)-C(19)	119.5(3)
C(17)-C(18)-C(21)	121.2(4)
C(19)-C(18)-C(21)	119.4(4)
C(20)-C(19)-C(18)	119.4(4)
C(20)-C(19)-H(19A)	120.3
C(18)-C(19)-H(19A)	120.3
C(15)-C(20)-C(19)	121.7(3)
C(15)-C(20)-H(20A)	119.2
C(19)-C(20)-H(20A)	119.2
F(3)-C(21)-F(1)	106.1(7)
F(3)-C(21)-F(2)	99.4(8)
F(1)-C(21)-F(2)	101.3(7)
F(3)-C(21)-C(18)	114.7(5)
F(1)-C(21)-C(18)	117.8(4)
F(2)-C(21)-C(18)	115.1(5)

Symmetry transformations used to generate equivalent atoms:

Table 4. Anisotropic displacement parameters ($Å^2$x 10^3) for **7g**. The anisotropic displacement factor exponent takes the form: $-2\pi^2[h^2 a^{*2}U^{11} + ... + 2 h k a^* b^* U^{12}]$

	U^{11}	U^{22}	U^{33}	U^{23}	U^{13}	U^{12}
S(1)	76(1)	86(1)	62(1)	-5(1)	45(1)	4(1)
F(1)	87(3)	443(11)	95(3)	-42(4)	64(2)	36(4)
F(2)	125(4)	418(12)	269(7)	-200(8)	159(5)	-127(6)
F(3)	402(12)	268(9)	535(15)	250(10)	432(13)	252(9)
N(1)	32(1)	40(1)	40(1)	-2(1)	18(1)	-4(1)
N(2)	34(1)	44(2)	41(1)	-5(1)	20(1)	-3(1)
N(3)	32(1)	38(2)	43(1)	-4(1)	17(1)	-4(1)
N(4)	36(1)	41(2)	47(1)	-2(1)	22(1)	-4(1)
C(1)	55(2)	61(2)	44(2)	-2(2)	26(2)	3(2)
C(2)	38(2)	44(2)	41(2)	3(1)	21(1)	3(1)
C(3)	44(2)	80(3)	54(2)	-18(2)	29(2)	-8(2)
C(4)	36(2)	65(2)	52(2)	-14(2)	24(2)	-4(2)
C(5)	34(2)	45(2)	36(1)	2(1)	16(1)	5(1)
C(6)	34(2)	46(2)	43(2)	-9(1)	18(1)	-3(1)
C(7)	38(2)	38(2)	35(1)	1(1)	16(1)	3(1)
C(8)	30(2)	36(2)	36(1)	2(1)	14(1)	1(1)
C(9)	46(2)	44(2)	62(2)	-10(2)	30(2)	-12(2)
C(10)	27(2)	41(2)	37(1)	3(1)	14(1)	0(1)
C(11)	34(2)	40(2)	40(2)	-1(1)	18(1)	4(1)
C(12)	43(2)	48(2)	43(2)	-5(1)	25(1)	-2(2)
C(13)	39(2)	44(2)	39(2)	6(1)	21(1)	2(1)
C(14)	44(2)	55(2)	66(2)	-3(2)	30(2)	-10(2)
C(15)	36(2)	44(2)	43(2)	4(1)	22(1)	3(1)
C(16)	38(2)	47(2)	42(2)	-3(1)	19(1)	-2(1)
C(17)	46(2)	55(2)	45(2)	-8(2)	23(2)	0(2)
C(18)	45(2)	69(3)	58(2)	-1(2)	34(2)	0(2)
C(19)	28(2)	111(4)	51(2)	-11(2)	16(2)	-1(2)
C(20)	40(2)	89(3)	42(2)	-10(2)	18(2)	0(2)
C(21)	42(2)	106(4)	77(3)	-19(3)	33(2)	9(3)

Table 5. Hydrogen coordinates (x 10^4) and isotropic displacement parameters ($Å^2$x 10^3) for**7g**.

	x	y	z	U(eq)
H(1)	11160	238	1286	62
H(3)	13290	1494	3827	68
H(4)	14258	928	2875	59
H(6A)	10219	-619	2355	49
H(9A)	12428	3409	3754	72
H(9B)	12233	3561	4671	72
H(9C)	11543	4384	3720	72
H(12A)	9266	3541	5638	51
H(14A)	7648	-1015	3460	79
H(14B)	6860	-192	3704	79
H(14C)	7683	-1165	4488	79
H(16A)	8371	2708	6646	50
H(17A)	7177	3220	7167	57
H(19A)	5086	2159	4583	77
H(20A)	6279	1673	4049	68

Table 6.Torsion angles [°] for **7g**.

C(4)-S(1)-C(1)-C(2)	-1.1(3)
S(1)-C(1)-C(2)-C(3)	0.6(5)
S(1)-C(1)-C(2)-C(5)	-175.3(3)
C(1)-C(2)-C(3)-C(4)	0.4(5)
C(5)-C(2)-C(3)-C(4)	176.2(3)
C(2)-C(3)-C(4)-S(1)	-1.1(5)
C(1)-S(1)-C(4)-C(3)	1.2(3)
C(8)-N(1)-C(5)-C(6)	0.4(4)
C(9)-N(1)-C(5)-C(6)	-167.3(3)
C(8)-N(1)-C(5)-C(2)	-176.7(3)
C(9)-N(1)-C(5)-C(2)	15.6(5)
C(1)-C(2)-C(5)-N(1)	-149.6(3)
C(3)-C(2)-C(5)-N(1)	35.1(5)

C(1)-C(2)-C(5)-C(6)	33.9(5)
C(3)-C(2)-C(5)-C(6)	-141.4(4)
N(1)-C(5)-C(6)-C(7)	-0.8(4)
C(2)-C(5)-C(6)-C(7)	176.1(3)
C(10)-N(3)-C(7)-C(6)	-179.5(3)
C(10)-N(3)-C(7)-C(8)	-1.6(4)
C(5)-C(6)-C(7)-N(3)	179.0(3)
C(5)-C(6)-C(7)-C(8)	0.9(4)
C(11)-N(2)-C(8)-N(1)	-178.9(3)
C(11)-N(2)-C(8)-C(7)	1.1(5)
C(5)-N(1)-C(8)-N(2)	-179.8(3)
C(9)-N(1)-C(8)-N(2)	-11.5(5)
C(5)-N(1)-C(8)-C(7)	0.2(3)
C(9)-N(1)-C(8)-C(7)	168.5(3)
N(3)-C(7)-C(8)-N(2)	1.0(5)
C(6)-C(7)-C(8)-N(2)	179.3(3)
N(3)-C(7)-C(8)-N(1)	-179.0(3)
C(6)-C(7)-C(8)-N(1)	-0.6(4)
C(7)-N(3)-C(10)-N(4)	178.9(3)
C(7)-N(3)-C(10)-C(11)	0.3(5)
C(13)-N(4)-C(10)-N(3)	-179.3(3)
C(14)-N(4)-C(10)-N(3)	13.6(5)
C(13)-N(4)-C(10)-C(11)	-0.5(3)
C(14)-N(4)-C(10)-C(11)	-167.5(3)
C(8)-N(2)-C(11)-C(10)	-2.4(4)
C(8)-N(2)-C(11)-C(12)	180.0(3)
N(3)-C(10)-C(11)-N(2)	1.9(5)
N(4)-C(10)-C(11)-N(2)	-176.9(3)
N(3)-C(10)-C(11)-C(12)	-180.0(3)
N(4)-C(10)-C(11)-C(12)	1.2(4)
N(2)-C(11)-C(12)-C(13)	176.5(3)
C(10)-C(11)-C(12)-C(13)	-1.4(4)
C(11)-C(12)-C(13)-N(4)	1.2(4)
C(11)-C(12)-C(13)-C(15)	-173.6(3)
C(10)-N(4)-C(13)-C(12)	-0.4(4)
C(14)-N(4)-C(13)-C(12)	165.5(3)
C(10)-N(4)-C(13)-C(15)	174.6(3)
C(14)-N(4)-C(13)-C(15)	-19.4(5)

C(12)-C(13)-C(15)-C(20)	138.0(4)
N(4)-C(13)-C(15)-C(20)	-36.1(5)
C(12)-C(13)-C(15)-C(16)	-36.6(5)
N(4)-C(13)-C(15)-C(16)	149.3(3)
C(20)-C(15)-C(16)-C(17)	0.5(5)
C(13)-C(15)-C(16)-C(17)	175.3(3)
C(15)-C(16)-C(17)-C(18)	-0.8(6)
C(16)-C(17)-C(18)-C(19)	1.4(6)
C(16)-C(17)-C(18)-C(21)	-180.0(4)
C(17)-C(18)-C(19)-C(20)	-1.8(7)
C(21)-C(18)-C(19)-C(20)	179.5(5)
C(16)-C(15)-C(20)-C(19)	-1.0(6)
C(13)-C(15)-C(20)-C(19)	-175.6(4)
C(18)-C(19)-C(20)-C(15)	1.6(7)
C(17)-C(18)-C(21)-F(3)	108.4(9)
C(19)-C(18)-C(21)-F(3)	-73.0(10)
C(17)-C(18)-C(21)-F(1)	-17.5(9)
C(19)-C(18)-C(21)-F(1)	161.1(7)
C(17)-C(18)-C(21)-F(2)	-137.0(7)
C(19)-C(18)-C(21)-F(2)	41.6(9)

Symmetry transformations used to generate equivalent atoms:
